# Capsaicin Alleviates Autophagy‐Lysosomal Dysfunction via PPARA‐Mediated V‐ATPase Subunit ATP6V0E1 Signaling in 3xTg‐AD Mice

**DOI:** 10.1002/advs.202502707

**Published:** 2025-07-28

**Authors:** Haitao Yu, Jia Chen, Fangzhou Wang, Dongdong Jia, Shuguang Bi, Yuming Mao, Yu‐qi Zhang, Tian‐long Gao, Liu Yang, Jia‐xu Du, Zi‐han Zhou, Xi‐Chen Zhu, Gao‐shang Chai

**Affiliations:** ^1^ Department of Fundamental Medicine Wuxi School of Medicine Jiangnan University MOE Medical Basic Research Innovation Center for Gut Microbiota and Chronic Diseases School of medicine Jiangnan university Wuxi Jiangsu Province 214122 P. R. China; ^2^ Department of Neurology Affiliated Hospital of Jiangnan University 1000 Hefeng Road Wuxi Jiangsu Province 214000 PR China; ^3^ Department of Neurology Affiliated Mental Health Center of Jiangnan University Wuxi Jiangsu Province 214151 P. R. China; ^4^ Department of Neurology Affiliated Wuxi Clinical College of Nantong University Wuxi Jiangsu Province 214000 China

**Keywords:** Alzheimer's disease, ATP6V0E1, autophagy, capsaicin, PPARA, V‐ATPase

## Abstract

Autophagy‐lysosomal pathway deficits contribute to the accumulation of amyloid‐β (Aβ), Tau, and lipid droplets in Alzheimer's disease (AD). Capsaicin, a specific agonist of transient receptor potential vanilloid 1 (TRPV1), can improve cognitive function in AD patients, but the detailed mechanism is still unclear. Here, it is revealed that capsaicin ameliorated AD‐related pathology by activating peroxisome proliferator‐activated receptor alpha (PPARA/PPARα, a key regulator of lipid metabolism) to promote lipid metabolism and reverse autophagy‐lysosomal deficits. Molecular mechanism research found that capsaicin significantly activated the PPAR signaling pathway to promote lipid metabolism, with PPARA identified as the key transcription factor. In addition, capsaicin upregulated ATP6V0E1 (V‐ATPase V0 complex subunit e1, involved in lysosomal acidification) expression through PPARA, restoring V‐ATPase activity. This enhanced lysosomal acidification facilitated lipophagy (autophagic clearance of lipid droplets), while promoting the clearance of Aβ and Tau aggregates via the autophagy‐lysosomal pathway. Further, inhibition of ATP6V0E1 and PPARA expression blocked the effect of capsaicin on alleviating AD lipid pathology and cognitive deficits through autophagy‐lysosomal flux. Taken together, capsaicin promotes lipid metabolism, reduces lipid deposition, and attenuates AD‐related pathologies, while PPARA‐ATP6V0E1‐V‐ATPase signaling mediated autophagy‐lysosomal pathway plays a key role in this process.

## Graphical abstract

Capsaicin enhances lipid metabolism, reduces lipid deposition, and alleviates AD‐related pathologies. Capsaicin activates PPARA, promoting the expression of PPAR signaling pathway‐related genes and accelerating lipid metabolism. Capsaicin upregulates ATP6V0E1‐V‐ATPase‐mediated lysosomal acidification, enhancing lipid droplet clearance. PPARA is the key transcriptional factor mediating capsaicin's regulation of the ATP6V0E1‐V‐ATPase signaling pathway.

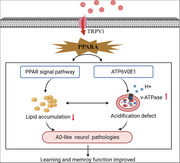

## 1 Introduction

Alzheimer's disease (AD) is the most common neurodegenerative disease, its main clinical manifestation is progressive memory loss^[^ ^1^ ^]^ and its pathological manifestations are amyloid (Aβ) plaque deposition and neurofibrillary tangles (NFTs) formed by hyperphosphorylated Tau.^[^ ^2^, ^3^ ^]^ In addition, lipid accumulation, one of the five most original AD pathologies reported by Alois Alzheimer, is closely associated with both familial and sporadic AD.^[^ ^4^, ^5^ ^]^ Lipid accumulation is positively correlated with both the Aβ plaque burden and Tau pathology level, indicating that lipid deposition may exacerbate Alzheimer's disease pathogenesis.^[^ ^6^ ^]^ Elevated cholesterol levels enhance the sequential cleavage of amyloid precursor protein (APP) by β‐secretase and γ‐secretase, thereby promoting Aβ production and aggregation.^[^ ^7^ ^]^ Additionally, conditioned medium from lipid‐laden microglia induces Tau phosphorylation and neurotoxicity in an APOE‐dependent manner.^[^ ^6^ ^]^ Therefore, targeted clearance of Aβ, hyperphosphorylated Tau, and lipid accumulations is an effective means of treating AD.

Lysosomal dysfunction is increasingly recognized as a pivotal contributor to AD pathogenesis,^[^ ^8^ ^]^ with impaired acidification emerging as a critical driver of cytotoxic cascades. Disrupted lysosomal acidification, which is mediated by defects in vacuolar ATPase (V‐ATPase) subunits, compromises hydrolase activity and leads to incomplete clearance of amyloid‐β (Aβ) aggregates and hyperphosphorylated Tau.^[^ ^9^ ^]^ Recent studies have shown that lysosomal acidification disorders induce the autophagic accumulation of Aβ in neurons, which produces PANTHOS (poisonous anthos (flower)), and is followed by changes in lysosomal membrane permeability, cathepsin release, lysosomal cell death, and subsequent extracellular release of Aβ culminating in extracellular plaque formation.^[^ ^10^, ^11^ ^]^ Recent studies have also revealed that persistent lysosomal damage induces inflammatory responses or facilitates the propagation of toxic protein aggregates, which exacerbates pronounced neuronal loss across multiple brain regions.^[^ ^12^ ^]^ Critically, lysosomal impairment disrupts lipid metabolism, which causes lipophagy failure and lipid accumulation.^[^ ^13^ ^]^ These findings highlight the urgent need for therapies that target lysosomal restoration to mitigate AD progression.

Capsaicin, a neuroactive phytochemical in chili peppers, exhibits multimodal anti‐ Alzheimer's disease properties through Aβ reduction, Tau phosphorylation suppression, and neuroinflammation alleviation in preclinical models.^[^ ^14^, ^15^, ^16^, ^17^, ^18^ ^]^ Notably, capsaicin regulates lipid homeostasis through multiple molecular targets, including uncoupling proteins (UCPs), ATP‐binding cassette (ABC) transporters, and peroxisome proliferation‐activated receptors (PPARs).^[^ ^19^ ^]^ Capsaicin upregulates PPARA expression through TRPV1 activation,^[^ ^20^ ^]^ and PPARA, as a key lipid metabolism regulator, enhances fatty acid oxidation gene expression and increases hepatic peroxisome abundance.^[^ ^21^ ^]^ Moreover, Yu et al. revealed that capsaicin enhances autophagic degradation pathways and improves microglial lipid metabolism in APOE4 transgenic mice, thereby attenuating neuroinflammation.^[^ ^22^ ^]^ Intriguingly, PPARA also governs lysosomal biogenesis and microglial autophagy, and it is generally considered a therapeutic target for AD.^[^ ^23^, ^24^, ^25^ ^]^ ATP6V0E1 is an important subunit of the V‐ATPase proton pump, and its expression is significantly reduced in lysosomal disorders,^[^ ^26^ ^]^ with its restoration shown to alleviate lysosomal dysfunction.^[^ ^26^ ^]^ Therefore, we hypothesize that capsaicin may not only directly mediate the regulation of lipid metabolism through PPARA signaling but also modulate ATP6V0E1 expression to influence lysosomal lipid degradation processes in AD.

In this study, we revealed that capsaicin could alleviate autophagy‐lysosomal dysfunction, which leads to reduced lipid droplet aggregation and AD pathology via the PPARA‐mediated V‐ATPase suite ATP6V0E1 signaling. Capsaicin promoted ATP6V0E1 expression through PPARA, thereby reversing V‐ATPase activity. Inhibition of ATP6V0E1 and PPARA expression blocked the ability of capsaicin to alleviate AD pathology and cognitive deficits through autophagy‐lysosomal flux. Our findings suggested that capsaicin could attenuate AD pathology by upregulating the PPARA‐ATP6V0E1‐V‐ATPase pathway, which also revealed a novel theoretical basis for capsaicin attenuating pathological progression and cognitive deficits in AD.

## 2 Results

### 2.1 Capsaicin Improves Spatial Memory Impairment in 3xTg‐AD Mice

**Figure 1:**
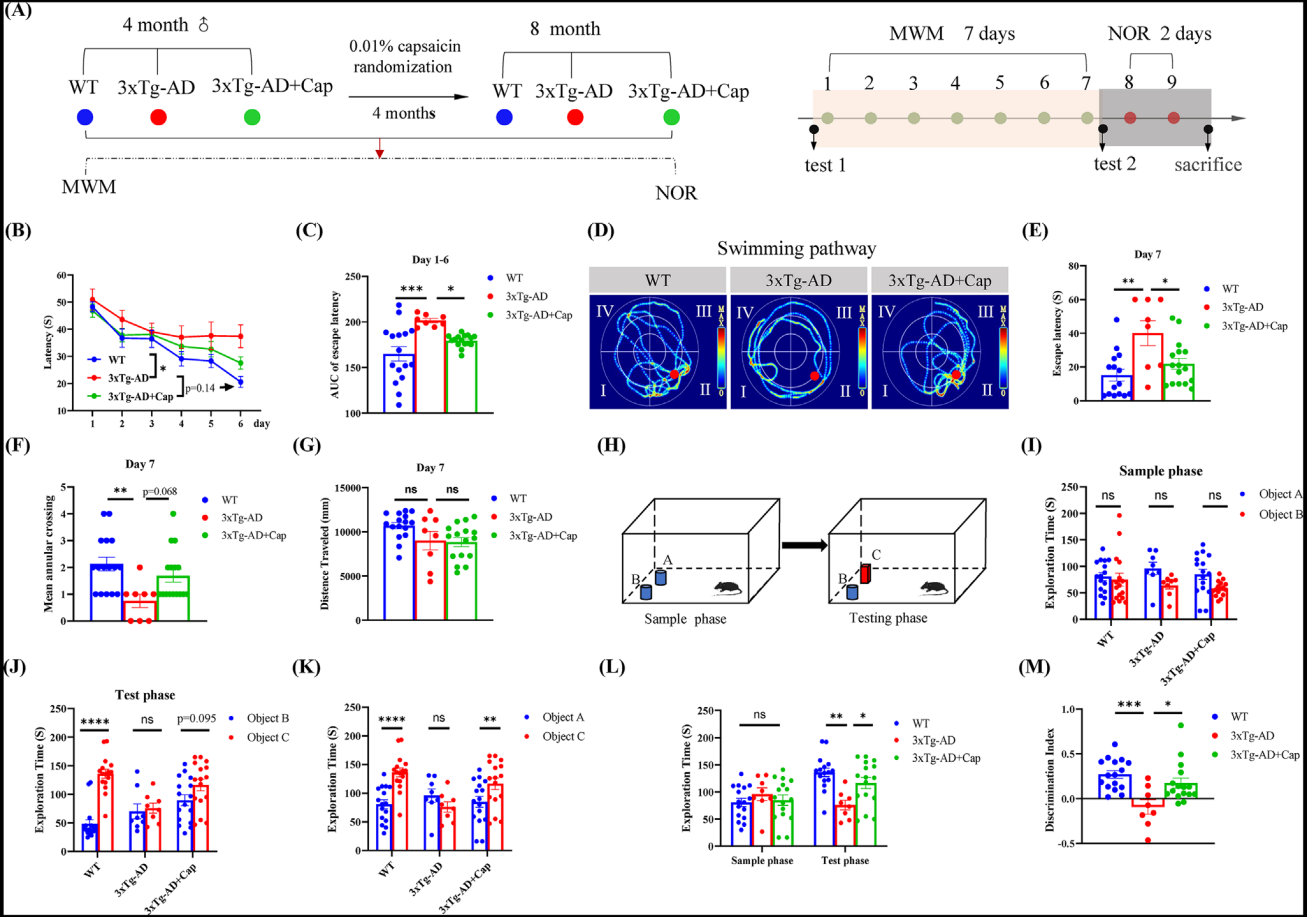
Capsaicin improves spatial memory impairment in 3xTg‐AD mice. A) Experimental workflow of the in vivo study. B–G) The Morris water maze (MWM) was used to detect the effect of capsaicin treatment on the spatial learning and memory of 3xTg‐AD mice fed with 0.01% capsaicin. B,C) Escape latency to the hidden platform in the training phase, and the AUC of escape latency. D) Swimming pathway traveled to locate the platform on day 7. E) The escape latency of day 7. F) The counts of the original position of the platform crossing on day 7. G) The distance traveled on day 7. H–M) The Novel‐object recognition test (NOR) was also used to assess the learning and memory ability of mice treated with 0.01% capsaicin. H) Schematic diagram of the replacement of the old to the new object. I) The change of exploration time of the three groups between object A and object B on sample phase. J) The change of exploration time of the three groups between object B and object C on test phase. K) The change of exploration time of the three groups between object A on the sample phase and object C on test phase. L) The exploration time for novel object between the sample phase and the test phase. M) Discrimination index of the new object. WT, *n* = 16, 3xTg‐AD, *n* = 8, 3xTg‐AD + Cap, *n* = 16. One‐way ANOVA followed by Tukey's post hoc test for (C, E, F, G, and M). Two‐way ANOVA test followed by Bonferroni's post hoc test for B, and I‐L. Data were shown as mean ± SEM. ^*^p < 0.05, ^**^p < 0.01, ^***^p < 0.001, ^****^p < 0.0001, ns, not significant.

The blood‐brain barrier (BBB) has limited the development of therapeutic drugs for AD. Capsaicin, as a compound that can effectively cross the blood‐brain barrier, has been widely used in research on AD‐related neurodegenerative diseases,^[^ ^27^ ^]^ and its absorption rate can reach 50%–90% after oral administration.^[^ ^28^ ^]^ After intravenous administration, five‐fold higher concentrations of the compound in the brain and spinal cord were reported than in the serum.^[^ ^27^ ^]^ Here, 4‐month‐old 3xTg‐AD mice were fed with a 0.01% capsaicin diet, while other mice were fed with a conventional diet based on the treatment regimen until 8‐month. During the treatment period, the body weights of the mice gradually increased in both the 3xTg‐AD + Cap group and the 3xTg‐AD group (Figure S1, Supporting Information), but there was no significant difference between the two groups, which was consistent with other research results.^[^ ^29^ ^]^ To determine whether capsaicin treatment improves the cognitive impairment of 3xTg‐AD mice, we performed Morris water maze (MWM) and new object recognition (NOR) experiments (**Figure** **1**A). The water maze was divided into four equal parts, which were named as four quadrants. An annular platform was a transparent circular platform with a diameter of 10 cm protruding 1.5 cm above the surface of the water and was placed in the center of the northwest (Session 2) quadrant (Figure S2A, Supporting Information).^[^ ^30^ ^]^ During the training period, the time spent searching for the platform was significantly shorter in all the groups during training (Figure 1B), and the time taken for 3xTg‐AD mice to find the platform was significantly longer than that of WT mice, whereas capsaicin treatment shortened the time needed to find the platform (Figure 1C), indicating that capsaicin improves the learning ability of the 3xTg‐AD mice. During the exploration period, the latency for 3xTg‐AD mice to first reach the platform was significantly longer than that of WT mice, whereas capsaicin treatment shortened the time to first cross the platform (Figure 1D,E), and the number of times that 3xTg‐AD mice crossed the annular platform was significantly decreased compared with that of WT mice and slightly increased after capsaicin treatment (Figure 1F). There was no statistically significant difference in the distance of mice swimming in the maze (Figure 1G). Additionally, the 3xTg‐AD mice spent significantly less time in the platform quadrant than the WT mice did during the test, whereas capsaicin treatment increased the time spent in the platform quadrant (Figure S2B, Supporting Information). The MWM experiment reveals that capsaicin improves the memory ability of AD model mice.

The NOR experiment revealed no statistically significant difference in the exploration time of object A and object B among all groups of mice in the sample phase (Figure 1I), although the 3xTg‐AD + Cap group exhibited comparable exploration of novel object C and familiar object B during testing (Figure 1J), this group exhibited significantly more exploration of new object C than of familiar object A (Figure 1K,L). In addition, the discrimination index of the mice in the 3xTg‐AD group in exploring old and new objects was significantly lower than that of WT mice, which could be markedly increased after capsaicin administration (Figure 1M). These results demonstrate that capsaicin improves the memory ability of AD model mice.

### 2.2 Capsaicin Activates PPAR Signaling Pathway to Promote Lipid Metabolism and Reduce Lipid Droplets Aggregation in the Hippocampus of 3xTg‐AD Mice

**Figure 2:**
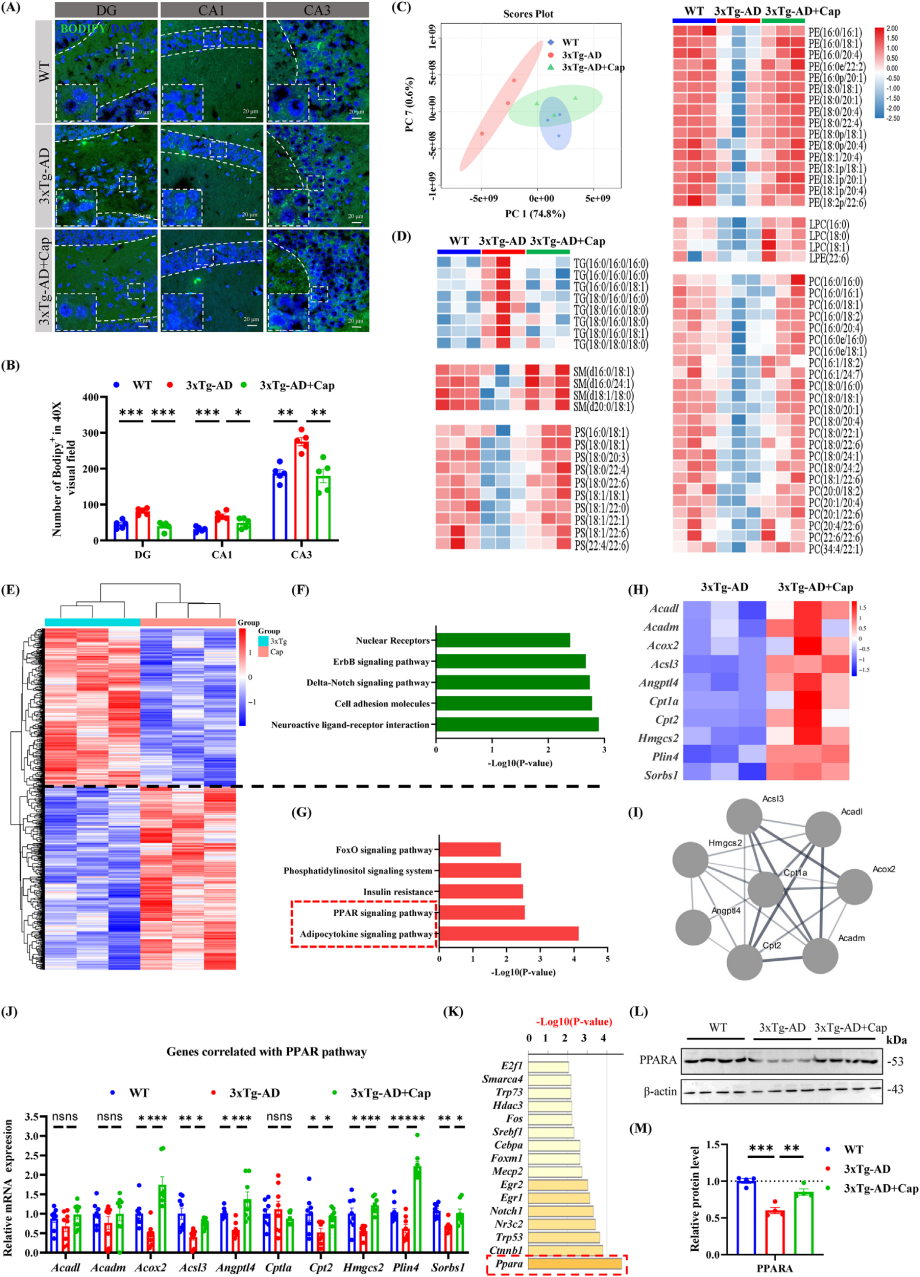
Capsaicin activates PPAR signaling pathway to promote lipid metabolism and reduce lipid droplets aggregation in the hippocampus of 3xTg‐AD mice. A and B) Immunofluorescence staining analysis of BODIPY (lipid droplets fluorescent dye) in the DG, CA1, and CA3 area of hippocampus in 3xTg‐AD mice treated with or without capsaicin and WT mice. A) Representative confocal images of BODIPY^+^ immunofluorescence labeling. B) Quantification of the number of BODIPY^+^ in the hippocampus of WT and 3xTg‐AD and 3xTg‐AD + Cap mice. *n* = 3/group, and 5 visual fields/group were imaged. C,D) Lipidomics results showed that triglyceride (TG) abnormalities, sphingomyelin (SM), and phospholipids (PS, PE, PC) were reduced in AD mice, and treatment with capsaicin ameliorated these phenomena. C) Principal component analysis (PCA) results demonstrated that the lipidomic data of AD mice were highly distinguishable from WT and capsaicin‐administered 3xTg‐AD mice, whereas 3xTg‐AD mice administered capsaicin partially crossed over with WT, suggesting that the lipidomics of AD mice tended to return to normal after capsaicin administration. D) The heatmap of changes in the content of different lipid components in the 3 groups of mice, *n* =  3/group. E) The heatmap of differentially expressed genes (DEGs, *p* < 0.05, fold change >1.5) in the hippocampus for 3xTg‐AD + Cap versus 3xTg‐AD mice, *n* = 3/group. The Z value of gene abundance was plotted in a red‐blue color scale, with red and blue indicating increased and decreased protein expression, respectively. F,G) KEGG enrichment analysis of decreased DEGs F), KEGG enrichment analysis of increased DEGs G). H,I) The heatmap H) and PPI network module I) of DEGs involved in the PPAR signaling pathway. J) The mRNA expression levels of 10 genes across three groups, *n* = 8/group. K) All DEGs were input into metascape online website (http://metascape.org/). and the transcription factors involved in regulation were predicted in the “Summary of enrichment analysis in TRRUST” module. L,M) The expression of PPARA in the hippocampus of WT mice, 3xTg‐AD mice, and 3xTg‐AD mice fed with capsaicin, *n* = 4/group. One‐way ANOVA followed by Tukey's post hoc test for (B, J, M). Data were shown as mean ± SEM. ^*^p < 0.05, ^**^p < 0.01, ^***^p < 0.001, ^****^p < 0.0001, ns, not significant.

Lipid accumulation, one of the five original AD pathologies reported by Alois Alzheimer, is closely associated with both familial and sporadic AD,^[^ ^4^, ^5^ ^]^ and targeted clearance of lipid droplets is essential for the treatment of AD. BODIPY, a lipophilic fluorescent probe, selectively targets intracellular neutral lipids—particularly those localized to lipid droplets—and has been extensively employed in studies of lipid metabolism, droplet biogenesis, and spatial lipid distribution. Here, we found that 3xTg‐AD exhibited lipid droplet aggregation via BODIPY staining and quantification number of BODIPY^+^ in 40X visual fields /of the DG, CA1, and CA3 regions, which was significantly reduced by the administration of capsaicin (**Figure** **2**A,B). In addition, lipidomic analysis revealed that capsaicin treatment caused the lipid metabolism in the hippocampus of 3xTg‐AD mice to be more similar to that of WT mice (Figure 2C). Moreover, triglyceride (TG) levels, one of the major components of lipid droplets, were significantly greater in the hippocampus of 3xTg‐AD mice than in that of WT mice, whereas capsaicin treatment significantly reduced TG levels (Figure 2D), suggesting that capsaicin may promote lipid droplet degradation. At the same time, several neuroprotective phospholipids, such as sphingomyelin (SM), phosphatidylserine (PS), phosphatidylethanolamine (PE), unsaturated phosphatidylcholine (LPC), and phosphatidylcholine (PC), substantially decreased in 3xTg‐AD, whereas their levels significantly increased after capsaicin treatment (Figure 2D). These data suggest that capsaicin may improve lipid homeostasis imbalance in the AD brain via promoting lipid degradation and metabolism.

Next, we found that capsaicin reversed gene expression in the hippocampus of 3xTg‐AD mice by transcriptome analysis (Figure 2E). The downregulated differentially expressed genes (DEGs) were significantly enriched in neuroactive ligand‐receptor interactions, cell adhesion molecules, delta‐Notch signaling pathway, ErbB signaling pathway, nuclear receptors, etc (Figure 2F), whereas the upregulated DEGs were significantly enriched in adipocytokine signaling pathway and PPAR signaling pathway, etc (Figure 2G). Further analysis revealed that the PPAR signaling pathway primarily includes *Acadl*, *Acadm*, *Acox2*, *Acsl3*, *Angptl4*, *Cpt1a*, *Cpt2*, *Hmgcs2*, *Plin4*, and *Sorbs1*, which form a closely related network module (Figure 2H,I). The qRT‐PCR validation revealed that PPAR signaling pathway‐related genes (*Acox2*, *Acsl3*, *Angptl4*, *Cpt2*, *Hmgcs2*, *Plin4*, *Sorbs1*) were significantly decreased in the hippocampus of 3xTg‐AD mice, which was notably reversed after the administration of capsaicin (Figure 2J). TRRUST (transcriptional regulatory relationships unraveled by sentence‐based text mining) analysis revealed that *Ppara* may be a key transcription factor in the action of capsaicin treatment, which is consistent with the reports that *Ppara* is a key regulator of the PPAR signaling pathway (Figure 2K). Finally, Western blotting (WB) validation experiments revealed that the PPARA level was significantly decreased in the hippocampus of 3xTg‐AD mice and markedly increased after capsaicin administration (Figure 2L,M).

These data suggest that capsaicin effectively reduces lipid accumulation in the hippocampus of 3xTg‐AD mice and that the mechanism involves the transcriptional expression of PPAR‐signaling pathway‐related genes regulated by PPARA, thereby reprogramming lipid metabolism.

### 2.3 Capsaicin Promotes Lipid Degradation by Enhancing the Autophagy‐Lysosomal Pathway

**Figure 3:**
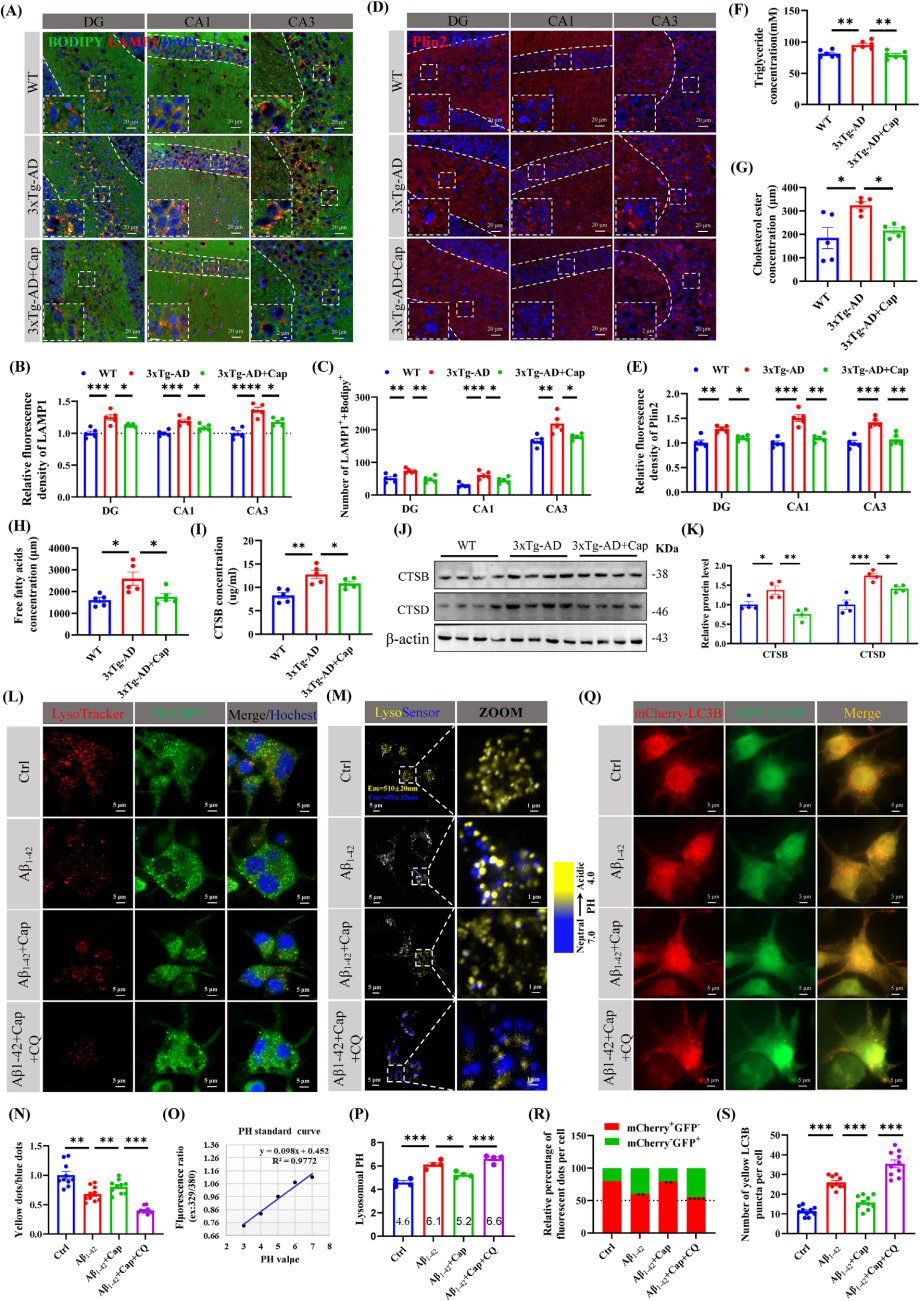
Capsaicin promotes lipid degradation by enhancing the autophagy‐lysosomal pathway. A,B) Characterization of the staining of BODIPY and LAMP1 (lysosome marker) in the hippocampus of 3xTg‐AD mice treated with or without capsaicin and WT mice via immunofluorescence, representative confocal images of BODIPY and LAMP1 immunofluorescence co‐labeling A), quantification of the relative fluorescence density of LAMP1^+^ in visual field B), quantification of the number of BODIPY^+^ and LAMP1^+^ in visual field, *n* = 3/group, 5 visual fields/group were imaged C). D,E) Immunohistochemical (IHC) analysis was performed to examine the distribution of Plin2 in hippocampal regions across the three experimental groups D), followed by quantitative statistical evaluation, n = 3/group, 5 visual fields/group were imaged E). F–H) Quantitative analysis of triglyceride (TG), *n* = 6/group F), cholesteryl ester (CE), *n* = 5/group G), and Free fatty acids (FFA) levels, *n* = 5/group H) was performed in hippocampal tissues from all three experimental groups. I) The concentration of cathepsin B (CTSB) in mouse hippocampal tissues was quantified by ELISA kit, *n* = 5/group. J,K) The protein expression levels of CTSB and CTSD were analyzed by Western blot J), followed by quantification, *n* = 4/group K). L) LysoTracker Red and BODIPY immunofluorescence were used to evaluate the lysosomal function and lipid droplets degradation. M,N) LysoSensor Yellow/Blue DND‐160 was used to distinguish acidic lysosomes (yellow puncta) from alkalinized lysosomes (blue puncta) in live cells M), followed by quantification, *n* = 10/group N). O,P) Lysosomal PH was calibrated using a standard curve generated with LysoSensor Yellow/Blue, *n* = 5/group O), and lysosomal PH of four groups, *n* = 4/group P). Q–S) The mCherry‐GFP‐LC3B plasmid and treated with Aβ_1‐42_, capsaicin and chloroquine (CQ), and the levels of autophagic flow were evaluated by quantifying GFP and mCherry fluorescence dots, representative confocal images of GFP and mCherry immunofluorescence co‐labeling Q), quantification of percentage of fluorescent dots/cell R), quantification of number of yellow LC3B puncta/cell, *n* = 10/group S). One‐way ANOVA followed by Tukey's post hoc test for (B, C, E, F, G, H, I, K, N, P, R, and S). Data were shown as mean ± SEM. ^*^p < 0.05, ^**^p < 0.01, ^***^p < 0.001, ^****^p < 0.0001, ns, not significant.

Lysosomal dysfunction is increasingly recognized as a pivotal contributor to AD pathogenesis,^[^ ^8^ ^]^ resulting in pathological lipid accumulation.^[^ ^31^ ^]^ Here, we examined the effect of capsaicin on the BODIPY staining and the lysosomal biomarker LAMP1 by co‐staining and found that the fluorescence intensity of LAMP1^+^ was increased in the hippocampus of 3xTg‐AD mice but decreased after capsaicin administration (**Figure** **3**A,B). In addition, the co‐localization of BODIPY^+^ and LAMP1^+^ was markedly increased in the hippocampus of 3xTg‐AD mice, but was decreased after capsaicin administration (Figure 3A,C). Triglyceride (TG) and cholesteryl esters (CEs) are the main components of lipid droplets.^[^ ^32^ ^]^ Insufficient fatty acid degradation may promote lipid droplet formation as a protective mechanism against free fatty acids (FFAs)‐induced lipotoxicity in AD.^[^ ^32^ ^]^ The level of perilipin 2 (PLIN2), a lipid‐droplet‐coating protein and marker, was elevated in the hippocampus of 3xTg‐AD mice but was reduced by capsaicin treatment, as shown by Western blotting (Figure S3A,B, Supporting Information) and immunofluorescence (Figure 3D,E). Similarly, we found that the levels of TG, CE, and FFAs were significantly elevated in the hippocampus of 3xTg‐AD mice, but were markedly reduced upon capsaicin treatment (Figure 3F–H), suggesting that capsaicin can effectively decrease lipid droplet accumulation. Cathepsins, key lysosomal enzymes responsible for intracellular waste and protein degradation, are tightly regulated by lysosomal PH.^[^ ^33^ ^]^ In the hippocampus of 3xTg‐AD mice, we observed significant upregulation of cathepsin B (CTSB) and cathepsin D (CTSD) expression (Figure 3I–K), which is consistent with reports of elevated cathepsin levels in AD patients and models, likely due to lysosomal acidification impairment triggered by compensatory cathepsin increases.^[^ ^33^, ^34^, ^35^ ^]^ Notably, capsaicin treatment restored CTSB and CTSD levels to those of wild‐type mice (Figure 3I–K). Taken together, these results suggest that capsaicin may promote the lipid droplets clearance via enhancing lysosomal function.

To investigate the direct effect of Aβ pathology on lipid deposition, and the effect of capsaicin on lipid autophagic degradation by enhancing lysosomal acidification, we treated N2a cells with Aβ_1‐42_ and capsaicin. Using LysoTracker Red and BODIPY probes to label acidic lysosomes and lipid droplets, respectively, we found that Aβ_1‐42_ treatment in N2a cells significantly reduced the number of red puncta (acidic lysosomes) and increased the number of green puncta (lipid droplets), while the phenotype was rescued by capsaicin treatment (Figure 3L; Figure S4A,B, Supporting Information). Conversely, chloroquine (CQ, 50 µm, 24 h) impaired lysosomal acidification,^[^ ^36^ ^]^ which led to a reduced LysoTracker Red signal and lipid droplets accumulation (Figure 3L; Figure S4A,B, Supporting Information). Lysosomes typically function at PH 4.2–5.3.^[^ ^37^ ^]^ To quantify lysosomal PH changes, we employed LysoSensor, a ratiometric probe that emits yellow fluorescence in acidic lysosomes and blue fluorescence in alkaline lysosomes.^[^ ^38^ ^]^ Aβ_1‐42_ treatment increased the lysosomal PH from 4.6 to 6.1 (Figure 3M–P), exceeding the optimal range for lysosomal degradation. Capsaicin treatment restored the PH to 5.2 and increased the number of acidic lysosomes, whereas CQ abolished this rescue effect (Figure 3M–P). In addition, Aβ_1‐42_ significantly inhibited autophagic flow (Figure 3Q–S). However, administration of capsaicin evidently improved autophagic flow (Figure 3Q–S). In sum, capsaicin promotes lipophagic degradation by enhancing lysosomal acidification.

To clarify the role of capsaicin in autophagy‐lysosomal pathway regulation, a series of molecular experiments were performed. First, qRT‐PCR and WB experiments were used to detect classic autophagy‐lysosome pathway‐related biomarkers. We found that LAMP1 expression was significantly increased in the hippocampus of 3xTg‐AD mice and decreased after the administration of capsaicin (Figure S5A–C, Supporting Information). At the protein level, LAMP1 is typically associated with lysosomal accumulation that results from impaired organelle turnover. However, the increase in LAMP1 at the transcriptional level may be related to the activation of lysosomal biogenesis induced by the transcription factor TFEB.^[^ ^39^ ^]^ The increase in the transcription of LAMP1 may be in response to lysosomal damage and accumulation, whereas the decrease in the transcription of LAMP1 may be due to the normal function of lysosomes without further lysosomal biogenesis. The expression of autophagic membrane formation‐related molecules ATG5, ATG7, and LC3B was significantly decreased in 3xTg‐AD mice, but was significantly increased after capsaicin administration, whereas p62 expression was significantly increased in the hippocampus of 3xTg‐AD mice and significantly decreased after capsaicin treatment (Figure S5A–C, Supporting Information). Capsaicin enhanced lysosomal activity and promoted the autophagy‐lysosomal pathway, where the p62 protein is recruited into autophagosomes and subsequently degraded, resulting in a decrease in the protein level of p62 (Figure S5B,C, Supporting Information). The increase in p62 mRNA level may be a compensatory response of cells to the decrease in p62 protein level during autophagy (Figure S5A, Supporting Information). Therefore, capsaicin promotes lipid degradation by enhancing the autophagy‐lysosomal pathway.

### 2.4 Capsaicin Increases ATP6V0E1‐Associated Lysosomal Function in 3xTg‐AD Mice

**Figure 4:**
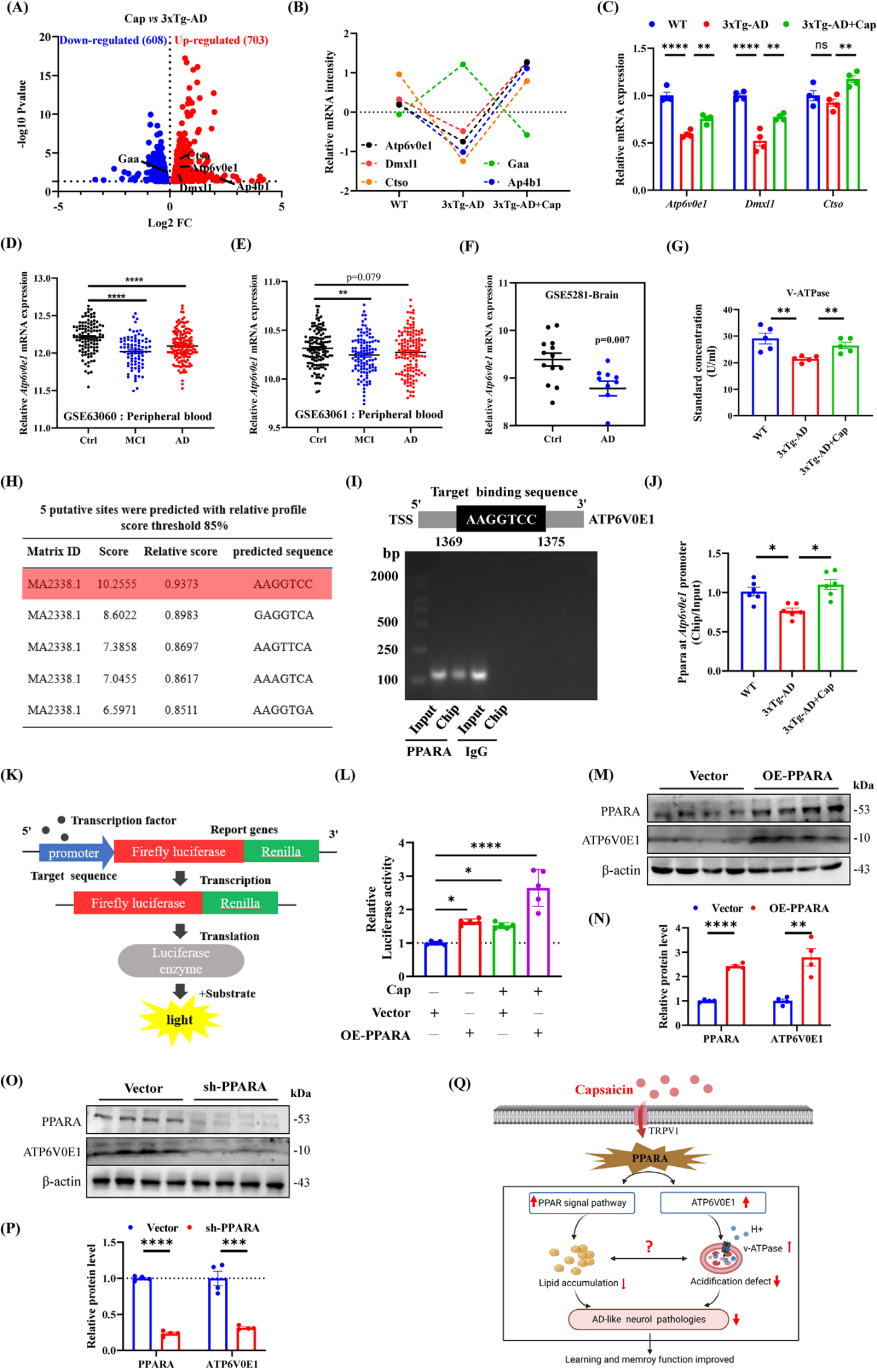
Capsaicin increases ATP6V0E1‐associated lysosomal function in 3xTg‐AD mice. A) Volcano plots of 3xTg‐AD + Cap versus 3xTg‐AD mice for lysosome‐related genes: the *x*‐axis represents the ratio in 3xTg‐AD + Cap versus 3xTg‐AD groups, and the *y*‐axis is the ‐log10‐transformed *p*‐value. The red color represented increased expression, and the blue color represented reduced expression. *n* = 3/group. B) Profile plot of mRNA expression of lysosome‐related genes, the dotted line connects the average abundance of the protein in the different animal groups, *n* = 3/group. C) The mRNA levels of lysosome‐related genes in the volcano plot were verified by qRT‐PCR analysis, *n* = 4/group. D–F) Relative mRNA expression levels of *Atp6v0e1* in peripheral blood of GSE63060, Ctrl *n* = 104, MCI *n* = 80, AD *n* = 145 D), peripheral blood of GSE63061, Ctrl *n* = 134, MCI *n* = 109, AD *n* = 139 E), and in brain of GSE5281, Ctrl *n* = 13, AD *n* = 10 F). G) The vacuolar ATPase (V‐ATPase) Elisa kit showed that V‐ATPase was decreased in the hippocampus of 3xTg‐AD mice, but reversed by capsaicin administration, *n* = 5/group. H) PPARA was predicted to have five sites of those were predicted with a relative profile score threshold 85% in the promoter region of ATP6V0E1 by JASPAR software. I,J) Chromatin immunoprecipitation (ChIP)‐qPCR showed that capsaicin promoted ATP6V0E1 transcription through PPARA, the image of agarose gel electrophoresis experiment of ChIP products I), the result of qPCR of the highest scoring prediction binding sequence, *n* = 6/group J). K,L) Dual‐luciferase reporter assay revealed that PPARA has at least one binding site to promote the transcription of ATP6V0E1, and capsaicin also promoted ATP6V0E1 transcription by activating PPARA‐ATP6V0E1 binding site, the pattern diagram of dual‐luciferase reporter experiment K), the ratio of relative light units of the two fluorescence intensities, *n* = 5/group L). M–P) Overexpression or knockdown of PPARA to further analyze the changes of ATP6V0E1 in protein level, when PPARA was overexpressed, the protein level of ATP6V0E1 was increased, *n* = 4/group M–N), while PPARA was knocked down, the protein level of ATP6V0E1 was decreased, *n* = 4/group O,P). (Q) Diagram of the amelioration of capsaicin‐mediated PPARA‐ATP6V0E1‐V‐ATPase pathway activation on lysosome function and lipid accumulations, and was created using BioRender (https://www.biorender.com/). Unpaired *t*‐test for (F, N, and P). One‐way ANOVA followed by Tukey's post hoc test for (C, D, E, G, J, and L). Data were shown as mean ± SEM. ^*^p < 0.05, ^**^p < 0.01, ^***^p < 0.001, ^****^p < 0.0001, ns, not significant.

Lysosomal acidification plays an important role in the ability of capsaicin to promote lipid reduction, but the specific mechanism is unclear. Interestingly, transcriptome analysis revealed that the lysosomal‐related genes *Atp6v0e1*, *Dmxl1*, *Ctso*, *Gaa*, and *Ap4b1* were dysregulated in the hippocampus of 3xTg‐AD mice, which could be reversed by the administration of capsaicin (**Figure** **4**A,B). Among them, *Atp6v0e1* controls lysosomal acidification,^[^ ^40^ ^]^ *Dmxl1* can induce V‐ATPase assembly,^[^ ^41^ ^]^ and *Ctso* is a hydrolytic enzyme that controls protein degradation.^[^ ^42^ ^]^ Consistent with the transcriptome data, the qRT‐PCR data revealed that *Atp6v0e1* and *Dmxl1* were significantly reduced in the hippocampus of 3xTg‐AD mice compared with WT mice, which could be reversed by capsaicin treatment (Figure. 4C). Moreover, the transcript level of Ctso did not change significantly in the 3xTg‐AD group, but increased after capsaicin administration (Figure 4C). Analysis of the GSE63060 dataset revealed significantly reduced *Atp6v0e1* expression in peripheral blood cells from both MCI and AD patients (Figure 4D), whereas the GSE63061 dataset showed a decrease in MCI (Figure 4E). Notably, *Atp6v0e1* expression was significantly reduced in AD hippocampal tissue (Figure 4F, GSE5281). In contrast, *Dmxl1* was significantly elevated in the AD brain, which was opposite to the mRNA data from the 3xTg‐AD mice (Figure S6, Supporting Information, GSE5281). Next, we detected V‐ATPase activity and found that capsaicin treatment significantly improved V‐ATPase activity in 3xTg AD mice (Figure 4G). Taken together, the significant dysregulation of genes related to lysosomal acidification, especially the decrease in ATP6V0E1, which is directly involved in hydrogen ion transport, induces autophagy‐lysosomal dysfunction in AD. Capsaicin ameliorates the process by upregulating ATP6V0E1.

Previous studies have shown that activation of PPARA‐mediated autophagy reduces Alzheimer's disease‐like pathology and cognitive decline in animal models,^[^ ^24^, ^43^ ^]^ suggesting that PPARA may be a key upstream transcription factor regulating the autophagy‐lysosomal pathway in AD. TRRUST analysis revealed that PPARA may be a key transcriptional factor involved in the action of capsaicin treatment (Figure 2K). However, the regulation of ATP6V0E1 expression by PPARA has not been reported. To further explore the mechanism by which capsaicin promotes ATP6V0E1 expression, we conducted upstream transcription factor prediction analysis. JASPAR transcription factor prediction analysis revealed 5 PPARA binding sites in the promoter region of ATP6V0E1, and the relative spectrum score threshold was 85%, which suggests that PPARA may be an upstream transcription factor of ATP6V0E1. (Figure 4H), and the protein expression of PPARA was consistent with expectation (Figure 2L,M). Chromatin immunoprecipitation (ChIP)‐qPCR experiment revealed that the enrichment of PPARA in the ATP6V0E1 promoter region was significantly reduced in the hippocampus of 3xTg‐AD mice, whereas the administration of capsaicin significantly promoted the enrichment of PPARA in the ATP6V0E1 promoter region (Figure 4I,J). The dual luciferase reporter gene assay results revealed that PPARA can activate ATP6V0E1 transcription (Figure 4K,L). Furthermore, overexpressing PPARA promoted ATP6V0E1 expression (Figure 4M,N), whereas knocking down PPARA inhibited ATP6V0E1 expression in N2a cells (Figure 4O,P). Based on these data, we propose the following scientific hypothesis: capsaicin activates the PPAR signaling pathway and the expression of ATP6V0E1 by promoting the expression of PPARA, thereby enhancing lipid metabolism and autophagy‐lysosome degradation of lipid droplets. This process ultimately slows AD‐like neuropathologies and improves learning and memory impairment in AD model mice (Figure 4Q).

### 2.5 Capsaicin Alleviates Autophagy‐Lysosomal Dysfunction via PPARA‐Mediated V‐ATPase Subunit ATP6V0E1 Signaling

**Figure 5:**
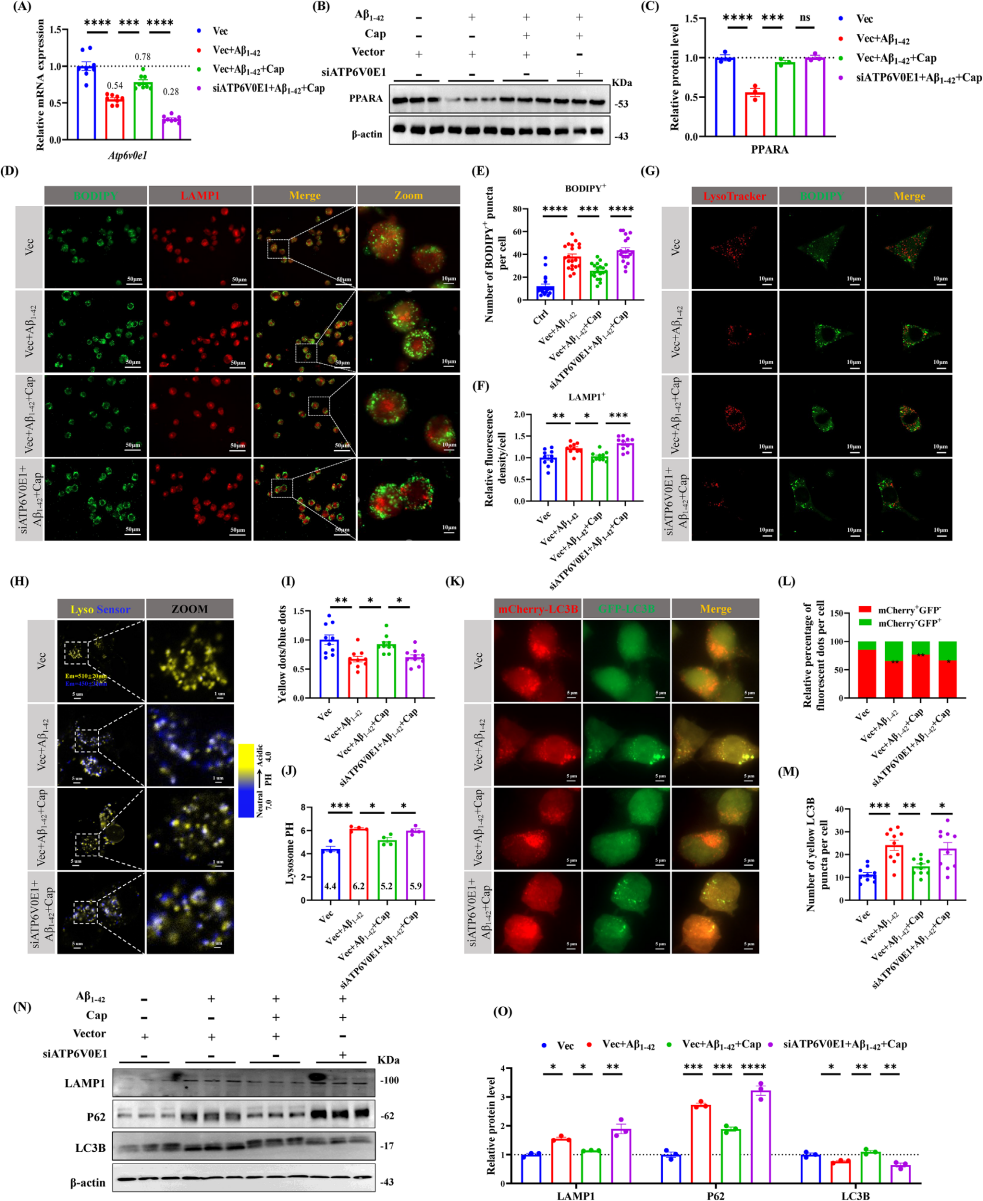
Inhibition of ATP6V0E1 expression blocked the effect of capsaicin on reducing lipid deposition through autophagy‐lysosomal flux. A) The mRNA levels of ATP6V0E1 in the four groups of cells were detected after finishing Vector or siATP6V0E1 transfection for 48 h, *n* = 8/group.  B,C) The protein level of PPARA after the transfection for 48 h, *n* = 3/group. D–F) Double immunofluorescence staining of LAMP1 and BODIPY confirmed that capsaicin improved autophagy and promoted lipid droplets degradation through ATP6V0E1 D), number of BODIPY^+^ particle per cell, *n* = 20/group E), relative fluorescence density/cell, *n* = 10/group F). G) LysoTracker Red and BODIPY immunofluorescence were used to evaluate the lysosomal function and lipid droplets degradation. H,I) LysoSensor Yellow/Blue DND‐160 was used to distinguish acidic lysosomes (yellow puncta) from alkalinized lysosomes (blue puncta) in live cells H), followed by quantification, *n* = 10/group I). J) Lysosomal PH of four groups, *n* = 4/group. K–M) The mCherry‐GFP‐LC3B plasmid and Vector or siATP6V0E1 RNA were transfected and subsequently treated with Aβ_1‐42_ and capsaicin, and the level of autophagic flow was evaluated by quantifying GFP and mCherry fluorescence signals, representative confocal images of GFP and mCherry immunofluorescence co‐labeling K), quantification of percentage of fluorescent dots/cell L), quantification of number of yellow LC3B dots/cell, *n* = 10/group M). N,O) The protein expression of autophagy‐lysosomal markers LAMP1, p62, LC3B, *n* = 3/group. One‐way ANOVA followed by Tukey's post hoc test for (A, C, E, F, I, J, L, M, and O). Data were shown as mean ± SEM. ^*^p < 0.05, ^**^p < 0.01, ^***^p < 0.001, ^****^p < 0.0001, ns, not significant.

To investigate the ability of ATP6V0E1 to promote lipid droplets degradation through the autophagolysosomal pathway, we used Aβ_1‐42_‐treated neuro‐2a cells (N2a) as an AD cell model to simulate the brain pathological environment of AD partially. Consistent with both human and animal AD data, we found that the expression of ATP6V0E1 was significantly decreased in AD cell models, significantly upregulated after capsaicin administration, and further decreased the expression of ATP6V0E1 by siRNA (**Figure** **5**A). In addition, WB experiments revealed that PPARA was significantly reduced in AD cells, and significantly upregulated after capsaicin administration, but siATP6V0E1 had no significant effect on the expression of *Ppara* (Figure 5B,C). Immunofluorescence revealed that Aβ_1‐42_ promoted the positive staining of BODIPY and LAMP1, and capsaicin reduced the positive staining of both, but siATP6V0E1 blocked the effect of capsaicin (Figure 5D,F). The co‐staining results of LysoTracker and BODIPY indicated that capsaicin improved lysosomal acidification ability and promoted lipid droplets reduction, whereas siATP6V0E1 inhibited the effect of capsaicin (Figure 5G). LysoSensor Yellow/Blue DND‐160 staining revealed that capsaicin significantly increased the proportion of yellow (acidic) lysosomes, whereas siATP6V0E1 reduced it (Figure 5H,I). Consistently, LysoSensor Yellow/Blue assays demonstrated that capsaicin markedly decreased lysosomal PH, whereas siATP6V0E1 increased it (Figure 5J). These data establish ATP6V0E1 as a critical mediator of the effects of capsaicin on lysosomal acidification. Moreover, Aβ_1‐42_ inhibited autophagy‐lysosomal flux, whereas capsaicin promoted autophagy‐lysosomal flux, which was inhibited again after ATP6V0E1 was downregulated (Figure 5K,M). Accordingly, the expression of autophagy‐lysosomal markers LAMP1 and p62, was significantly increased after Aβ_1‐42_ treatment, while LAMP1 and p62 were significantly decreased after capsaicin administration, but LAMP1 and p62, were increased again after further downregulation of ATP6V0E1 expression, however, LC3B expression was the opposite of that described above (Figure 5N,O). Together, these results indicate that capsaicin can reverse AD autophagy‐lysosomal disorders and reduce the number of lipid droplets via increasing ATP6V0E1 expression.

**Figure 6:**
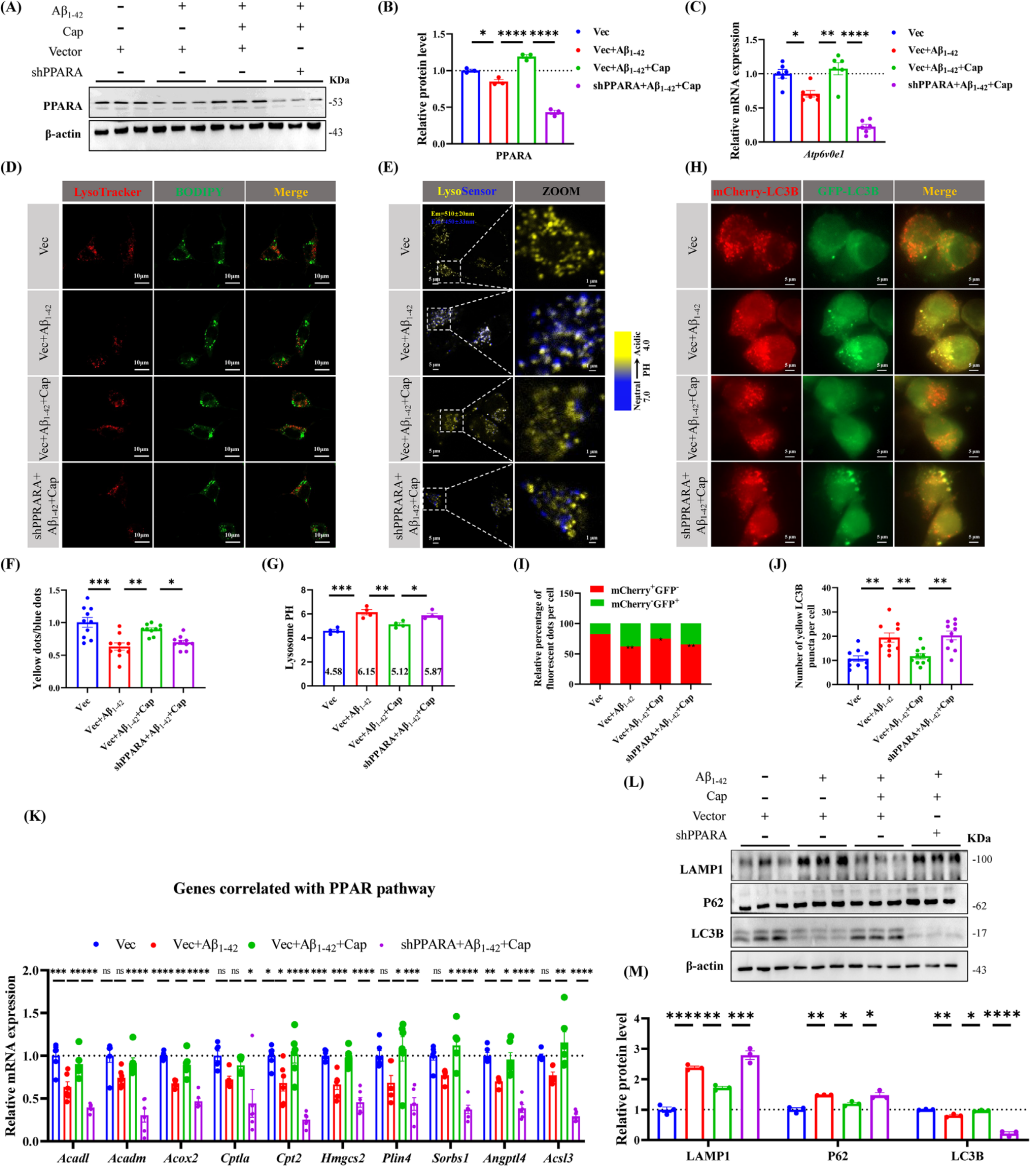
Inhibition of PPARA expression blocked the effect of capsaicin on reducing lipid deposition through autophagy‐lysosomal flux. A,B) The protein level of PPARA in the four groups after finishing the Vector or shPPARA transfection for 48 h, *n* = 3/group. C) The mRNA level of ATP6V0E1 after finishing the transfection for 48 h, *n* = 6/group. D) Immunofluorescence double labeling of LysoTracker Red and BODIPY. E,F) LysoSensor Yellow/Blue DND‐160 was used to distinguish acidic lysosomes (yellow puncta) from alkalinized lysosomes (blue puncta) in live cells (E), followed by quantification, *n* = 10/group F). G) Lysosomal PH of four groups, *n* = 4/group. H–J) The immunofluorescence of mCherry‐GFP‐LC3B plasimd among the four groups, representative confocal images of GFP and mCherry immunofluorescence co‐labeling H), quantification of percentage of fluorescent dots/cell I), quantification of number of yellow LC3B dots/cell, *n* = 10/group J). K) The relative mRNA levels of genes correlated with the PPAR pathway, *n* = 6/group. L,M) The protein expression of autophagy‐lysosomal markers LAMP1, p62, LC3B, *n* = 3/group. One‐way ANOVA followed by Tukey's post hoc test for (B, C, F, G, I, J, K, and M). Data were shown as mean ± SEM. ^*^p < 0.05, ^**^p < 0.01, ^***^p < 0.001, ^****^p < 0.0001, ns, not significant.

To investigate the regulation of the autophagy‐lysosomal pathway by influencing the expression of ATP6V0E1, we knocked down PPARA. Consistent with the AD animal data, we found that PPARA expression was significantly reduced in AD cell models, significantly upregulated after capsaicin administration, and reduced by shPPARA (**Figure** **6**A,B). Accordingly, the ability of capsaicin to promote *Atp6v0e1* expression was abolished upon PPARA knockdown (Figure 6C). Immunofluorescence revealed that Aβ_1‐42_ promoted positive BODIPY staining, and that capsaicin significantly decreased positive BODIPY staining, but downregulation of PPARA significantly increased the positive staining, at the same time, capsaicin effectively recovered the lysosomal acidic environment, which can be reversed by shPPARA (Figure 6D). LysoSensor Yellow/Blue DND‐160 staining revealed that shPPARA significantly attenuated the capsaicin‐induced increase in yellow (acidic) lysosomes (Figure 6E,F). Consistently, LysoSensor Yellow/Blue assays demonstrated that shPPARA markedly suppressed the PH‐lowering effects of capsaicin on lysosomes (Figure 6G). In addition, autophagy‐lysosomal flux was again blocked after downregulation of PPARA (Figure 6H,J). We tested the levels of genes correlated with the PPARA pathway, and the results revealed that capsaicin increased the mRNA levels of those genes, and this effect could be strikingly reversed by downregulation of PPARA (Figure 6K). Similarly, the expression of autophagy‐lysosomal markers LAMP1 and p62 increased again after further downregulating the expression of PPARA, and that of LC3B was the opposite of that described above (Figure 6L,M). Together, these results suggest that capsaicin alleviates autophagy‐lysosomal dysfunction and promotes lipid droplets degradation via PPARA‐mediated V‐ATPase subunit ATP6V0E1 signaling.

### 2.6 Capsaicin Ameliorates AD‐Related Pathology by Enhancing the Autophagy‐Lysosomal Pathway

To investigate the role of capsaicin in promoting the degradation of Aβ and Tau pathology through the autophagy‐lysosome pathway, we performed the following experiments: an immunofluorescence assay revealed that the size and percentage of 4G8^+^ staining area of Aβ pathological plaques were significantly increased in the hippocampus of 3xTg‐AD mice and decreased in 3xTg‐AD mice after capsaicin administration (Figure S7A–C, Supporting Information), the lysosomal biomarker LAMP1^+^ was significantly increased in the hippocampus of 3xTg‐AD mice but significantly decreased after capsaicin administration (Figure S7D, Supporting Information), and the number of cells with co‐localized of 4G8^+^ and LAMP1^+^ was significantly increased in the hippocampus of 3xTg‐AD mice, but it was decreased after capsaicin administration (Figure S7E, Supporting Information). The WB experiments revealed that the Aβ content was evidently increased in the hippocampus of 3xTg‐AD mice and decreased after capsaicin treatment (Figure S7F,G, Supporting Information), at the same time, we also tested the changes in Tau pathology via WB experiments, and the results revealed that pS262, pS214, pS396, and pS404 were significantly increased in the hippocampus of 3xTg‐AD mice and decreased after capsaicin administration (Figure S7F,G, Supporting Information). Taken together, these findings suggest that capsaicin promotes lipid droplets degradation by enhancing the autophagy‐lysosomal pathway and correspondingly reduces Aβ and Tau‐related pathology.

### 2.7 Capsaicin reduces Inflammatory Activation and Lipid Accumulation of Microglia and Astrocytes in 3xTg‐AD Mice

In addition, we found that microglia and astrocytes were significantly activated in the hippocampus of 3xTg‐AD mice, whereas microglia and astrocytes activation was significantly inhibited after administration of capsaicin (Figure S8A,B,D,E, Supporting Information). Accordingly, we found that the co‐localization of Iba1^+^ and BODIPY^+^ or GFAP^+^ and BODIPY^+^ cells was also significantly reduced after capsaicin administration (Figure S8A,C,D,F, Supporting Information). We also measured the protein levels of GFAP and Iba1, which were increased in the hippocampus of 3xTg‐AD mice but decreased after 0.01% capsaicin was given (Figure S8G,H, Supporting Information). Thus, capsaicin not only reduces lipid accumulation but also attenuates inflammatory activation in both microglia and astrocytes.

## 3 Discussion

Lipid accumulation, one of the five original AD pathologies reported by Alois Alzheimer, is closely associated with both familial and sporadic AD,^[^ ^4^, ^5^ ^]^ and targeted clearance of lipid droplets is essential for the treatment of AD. Autophagy dysfunction is an important pathophysiological mechanism of AD, and which leads to insufficient lipid droplet clearance, which further exacerbates brain neuronal damage and inflammatory response.^[^ ^44^ ^]^ Therefore, effective acceleration of lipid metabolism and lipid clearance can significantly reduce AD‐like neuropathologies and improve learning and memory. Here, we found that capsaicin can promote the expression of PPAR signaling pathway‐related genes by activating PPARA, thereby accelerating lipid metabolism. Notably, PPARA also governs lysosomal biogenesis,^[^ ^23^, ^24^, ^25^ ^]^ whereas ATP6V0E1 is indispensable for lysosomal acidification.^[^ ^26^ ^]^ Through dual luciferase reporter assays and functional molecular studies, we established PPARA as the key transcriptional regulator of ATP6V0E1. Mechanistically, PPARA‐driven ATP6V0E1 promotes capsaicin‐induced lipid droplet clearance via V‐ATPase‐dependent potentiation of the autophagy‐lysosomal pathway. Enhanced lipid metabolism and degradation of lipid droplets can reduce lipid accumulation, thereby slowing down lysosomal dysfunction. Thus, capsaicin reduced lipid accumulation and alleviated AD‐related pathological changes, in which the PPARA‐ATP6V0E1‐V‐ATPase signaling mediated autophagy‐lysosomal pathway played a key role in this process (Figure 4Q).

Lipid accumulation precedes Aβ and Tau pathology, worsens with age, and is closely associated with the immune response and synaptic dysfunction.^[^ ^45^ ^]^ In particular, lipid metabolism disorders lead to lipid accumulation, which induces inflammatory responses in microglia, and affects the formation of presynaptic membranes and the energy supply.^[^ ^44^ ^]^ Here, the expression of the PPAR signaling pathway‐related genes *Acadl, Acadm, Acox2, Acsl3, Angptl4, Cpt1a, Cpt2, Hmgcs2, Plin4, and Sorbs1* was significantly reduced in 3xTg AD mice, but significantly increased after capsaicin administration. Capsaicin can enhance the expression of PPAR signaling‐related genes, promote lipid metabolism, and reduce lipid demand and energy supply in neurons and synapses. Specifically, Hmgcs2 overexpression promoted ketone body metabolism and alleviated AD‐related pathologies by regulating fatty acid β‐oxidation.^[^ ^46^, ^47^ ^]^ The carnitine palmitoyl transferase (CPT) genes Cpt1a and Cpt2 are located in the outer and inner membranes of mitochondria, and participate in the regulation of acylcarnitines and amino acids in AD, affecting synapses and neuroimmune regulation.^[^ ^48^, ^49^ ^]^ Correspondingly, capsaicin significantly reduced the inflammatory response in microglia and astrocytes. Methylation of the Sorbs1 gene affects neuronal activity and exacerbates cognitive impairment in AD patients.^[^ ^45^ ^]^ Therefore, capsaicin activates the PPAR signaling pathway to promote lipid metabolism and regulate synaptic and neuroimmune functions.

Blocking the autophagy‐lysosome pathway via lipid aggregation promotes neuroinflammation and a series of AD‐like neurological damage, whereas autophagy‐lysosome dysfunction reduces lipid droplets clearance, further exacerbating lipid aggregation, thus forming a vicious cycle of lipid aggregation/autophagy‐lysosomal pathway.^[^ ^31^, ^50^ ^]^ Lysosomal hydrolases need to be in an acidic environment to play a role in digestion and decomposition, thereby ensuring the smooth autophagic lysosome pathway: this acidic environment is achieved mainly by the V‐ATPase pumping H^+^ ions from the cytoplasm into the lysosome.^[^ ^51^ ^]^ Once lysosome acidification is hindered, the garbage decomposition pipeline stops, causing many garbage intermediate products to form and accumulate in the lysosome, including Aβ, Tau protein, and lipid droplets.^[^ ^8^, ^11^, ^31^, ^50^ ^]^ Importantly, lysosomes are the targets of AD pathogenic gene products and risk factors, including the pathogenic amyloid precursor protein (APP) metabolites APP‐βCTF and Aβ encapsulated in endosomes and autophagosomes.^[^ ^8^, ^52^ ^]^ ATP6V0E1 is an important V0 subunit of the V‐ATPase proton pump, and its expression is significantly reduced in lysosomal disorders.^[^ ^26^ ^]^ However, its changes in AD and specific mechanisms of action on autophagic lysosomes are not yet clear. In this study, we found that ATP6V0E1 was significantly decreased in AD patients and 3xTg‐AD mice and that the autophagosome was impaired. Capsaicin significantly increased the level of ATP6V0E1 and enhanced the autophagosome pathway, whereas siATP6V0E1 abolished the effect of capsaicin on autophagic flow. These results suggest that ATP6V0E1 plays a crucial role in capsaicin‐mediated improvements in the autophagy lysosomal pathway. Previous studies have shown that capsaicin can also promote the autophagic degradation of lipids by promoting the expression of autophagy‐related ATG5 and ATG7.^[^ ^53^ ^]^ We observed the upregulation of ATG5 and ATG7, which may cooperatively enhance ATP6V0E1‐mediated lysosomal function to promote lipid degradation, the underlying mechanisms require further investigation.

To further elucidate the key upstream molecules of capsaicin in reducing lipid aggregation and improving autophagy, we combined RNA‐seq analysis with capsaicin treatment to identify the potential upstream transcription factor PPAR. PPARA, a member of peroxisomes proliferator‐activated receptors (PPARs), is considered as an important regulator of lipid metabolism, and as a ligand‐activated transcription factor, it not only increases the expression of fatty acid oxidation genes, but also increases the abundance of peroxisomes in the liver.^[^ ^21^ ^]^ Growing evidences have shown that PPARA effectively enhances microglial autophagy under physiological and pathological conditions, and that it is generally considered a therapeutic target for AD.^[^ ^23^, ^24^, ^25^ ^]^ Specifically, PPARA can enhance microglial phagocytosis of Aβ,^[^ ^24^ ^]^ and activate TFEB to regulate lysosome generation, enhance autophagy, and promote Aβ degradation.^[^ ^23^, ^24^, ^25^ ^]^ Here, capsaicin promotes the expression of PPARA and lipid metabolism by activating the PPAR signaling pathway, and controls lysosomal acidification via promoting the transcription of *ATP6V0E1* expression, thereby enhancing the clearance of lipid droplets by autophagy. Thus, PPARA is crucial for capsaicin to promote lipid droplets clearance and reduce lipid aggregation, and the specific mechanism involves the activation of the PPAR signaling pathway and the regulation of ATP6V0E1‐V‐ATPase signaling in the autophagy‐lysosomal pathway.

Previous studies have shown that capsaicin can alleviate cognitive and synaptic plasticity impairment by activating TRPV1, further inhibiting APP processing and Aβ deposition, and AMPAR endocytosis in AD mouse models.^[^ ^54^, ^55^ ^]^ Moreover, capsaicin shifted amyloid precursor protein (APP) processing toward α‐cleavage and precluded Aβ generation by promoting the maturation of a disintegrin and metalloproteinase 10 (ADAM10), attenuating Tau hyperphosphorylation, neuroinflammation, neurodegeneration, and cognitive deficits in APP/PS1 mice.^[^ ^56^ ^]^ In addition, Yu et al. reported that capsaicin could activate TRPV1, promote the autophagic degradation pathway, further improve microglial lipid deposition, slow down neuroinflammation in APOE4 mice,^[^ ^22^ ^]^ and improve microglial metabolic reprogramming in APP/PS1 mice.^[^ ^57^ ^]^ In addition, capsaicin can improve glucose metabolism and mitophagy in microglia through the AKT‐mTOR‐HIF‐1α pathway, thereby alleviating the pathology of Parkinson's disease (PD) in model mice.^[^ ^58^ ^]^ The above evidence suggests that capsaicin plays an important role in slowing down AD/PD‐like pathology and reprogramming lipid and glucose metabolism processes in the brain, where the autophagy pathway may be essential. Our study highlights the key role of capsaicin in regulating lipid metabolism in AD brain through the PPAR signaling pathway and the PPARA‐ATP6V0E1‐V‐ATPase mediated autophagy‐lysosomal pathway.

In sum, our results provide strong evidence that capsaicin protects against lipid aggregation‐related neurodegeneration in 3xTg‐AD mice. Specifically, we found that the PPAR signaling pathway and ATP6V0E1‐V‐ATPase‐mediated lysosomal acidification were enhanced, thereby promoting lipid metabolism and degradation, and thus reducing AD‐like neuropathologies and improving learning and memory functions. Our data may help guide capsaicin intervention in patients with cognitive impairment caused by lipid metabolism disorders, ultimately reducing the incidence and slowing the progression of AD.

## 4 Experimental Section

### Animals and Drug Administration

Triple transgenic AD male mice (3xTg‐AD) (Stock No: 34830, 129S4.CgTg [APPSwe, tauP301L] 1LfaPsen1tm1Mpm/Mmjax) and wild‐type mice were a gift from Prof. Xifei Yang (Shenzhen Center for Disease Control and Prevention). The experimentation was authorized by the Animal Ethics Committee of Jiangnan University (JN. No20240315m0360701[100]). The area in which they were fed maintained a temperature range of 22–26 °C, while the relative humidity was kept between 50% and 60%, and adequate food and water were ensured, with 12 h light‐dark alternating. It is important to note that all experiments conducted with these mice adhere to the ethical considerations and animals' well‐being. All mice were given a normal diet. Starting at 4 months of age, 0.01% capsaicin (T1062, TargetMol, China) was added to the capsaicin group ^[^ ^56^ ^]^ for 4 months, while the WT and 3xTg‐AD groups were fed with a normal diet. Mice all sacrificed at 8 months of age. The daily intake of capsaicin was 30 mg kg^−1^ according to the food intake and body weights of the mice.

### Morris Water Maze Test

The Morris water maze test was used to evaluate the spatial learning and memory ability of mice. Before the start of the experiment, the mice were placed in a water maze with a diameter of 120 cm and swam freely for 2 min to familiarize themselves with the maze environment. The experiment lasted for seven days, and was scheduled from 8:00 to 18:00 every day. Each mouse was placed in water from four different quadrants and a 15 min gap between each trial for six days, and given certain cues around, recording the time they found the annular to evaluate their learning ability. On the seventh day, the platform was removed for exploration experiments, the number of times the annulus was crossed, the distance mice swam, and the residence time of the mice in different annulus areas in the maze during the test time were recorded to evaluate the mice's memory ability. In this experiment, the standard for finding the platform was for the mice to climb onto the platform and stay on it for more than 1 s. The WMT‐100 system (WMT‐100, TECHMAN, China) was used to capture movement images and experimental data from mice.

### Novel‐Object Recognition Test

The general procedure consisted of three different phases: a familiarization phase, a sample phase, and a test phase, which was consistent with other research methods.^[^ ^59^ ^]^ At the familiarization phase, mice were placed in a 60 × 60 × 50 cm^3^ box for 10 min to familiarize the environment. At the sample phase (24 h later), two identical cylinders with a base diameter of 6 cm and a height of 20 cm, A and B, were placed in each corner of the box, and the mice were allowed to explore it freely for 10 min. The next day of the test phase, the A cylinder in one corner of the box was replaced by a cuboid C with a length of 4 cm, a width of 2 cm, and a height of 10 cm, while the cylinder B stayed at the same position, and the mice were allowed to explore it freely for 10 min. The time spent in every object area for exploring the objects was recorded during the experiment. Each trial was wiped with 75% alcohol at the end to eliminate odor effects. The discriminant index was calculated using the formula D.I. = (Tnovel – Tfamiliar) / (Tnovel + Tfamiliar) × 100%. The Morris water maze's camera (WMT‐100, TECHMAN, China) setup to track the mice's effective exploration time was additionally used.

### RNA‐Sequencing Analysis

Transcriptomic analysis was performed to comprehensively investigate differentially expressed genes in the 3xTg‐AD + Cap versus 3xTg‐AD comparison groups, including RNA purification, reverse transcription, library construction, and sequencing. These procedures were conducted by Shanghai Majorbio BioPharm Technology Co., Ltd. The differentially expressed genes (DEGs) were defined as *p*‐value < 0.05 and fold change (FC) >1.1. The biological functions and pathways related to the differentially expressed genes were further analyzed by Kyoto Encyclopedia of Genes and Genomes (KEGG) and the WEB‐based Gene Set Analysis Toolkit (http://www.webgestalt.org).

### Lipid Extraction and LC/MS Analysis

For lipid extraction from fresh hippocampal tissue, 300 µL of 100% methanol (G75851B, Tansoole, China) was added and vortexed thoroughly followed by addition of 1 mL methyl tert‐butyl ether (MTBE) (G28130B, Tansoole, China) with continuous agitation at room temperature for 1 h, after which 250 µL of deionized water was added and mixed vigorously before allowing the mixture to stand for 10 min and subsequent centrifugation at 6000 rpm for 3 min, the resulting supernatant was then evaporated to complete dryness under nitrogen stream using a Reacti‐Vap nitrogen evaporator (TS‐18825, Thermo Fisher Scientific, USA), and the dried lipid residue was reconstituted in 50 µL of methanol‐chloroform (1:1, v/v) mixture followed by addition of 200 µL of solvent mixture (2B + A) [Solvent A consisted of 10 mM ammonium acetate (1 085 756, Leyan, China) in 90:10 (v/v) isopropanol/acetonitrile (G80988B, Tansoole, China)], [Solvent B was 10 mm ammonium acetate in 60:40 (v/v) acetonitrile/water] before final centrifugation at 15000 rpm for 15 min at 4 °C, from the cleared supernatant, 180 µL was carefully transferred to LC/MS vials while equal 20 µL aliquots from nine individual samples were added to create a quality control (QC) sample for monitoring LC/MS system performance (Vanquish Q Exactive Plus system, Thermo Fisher Scientific, USA), with all acquired mass spectrometry data being processed and analyzed using LipidSearch software (version 4.2, Thermo Fisher Scientific, USA) for comprehensive lipid identification and quantification.

### Free Fatty Acids level

The levels of free fatty acids in mice hippocampal tissues were determined using the Free Fatty Acids Assay Kit (S0215S, Beyotime, China), with hippocampal tissues homogenized in isopropanol using a tissue homogenizer (issuelyser‐24L, Jingxin, China) followed by centrifugation at 12,000 g for 5 min at 4 °C to collect the supernatant, the samples were then diluted at least five‐fold with assay buffer and 20 µL of the diluted samples were added to each well of a 96‐well plate along with isopropanol‐containing assay buffer to adjust the final volume to 50 µL per well, after which 2 µL of Enzyme mixture B was added to each well and incubated at 37 °C in the dark for 30 min, subsequently, 50 µL of free fatty acids detection working solution was added to each well and mixed thoroughly followed by another 37 °C incubation in the dark for 30 min, with the absorbance at 570 nm measured using a microplate reader (Synergy H4, Bio Tek, USA) to establish a standard curve and calculate the concentration of free fatty acids in the samples based on the standard curve.

### Triglyceride Level

The triglyceride levels in mice hippocampal tissues were quantified using a commercial Triglyceride Assay Kit (S0219S, Beyotime, China) according to the manufacturer's protocol, with hippocampal tissues homogenized in isopropanol using a tissue homogenizer (tissuelyser‐24L, Jingxin, China) followed by centrifugation at 12000 × g for 5 min at 4 °C to obtain the supernatant, which was then diluted at least five‐fold with assay buffer before loading 20 µL of the diluted sample into each well of a 96‐well plate along with isopropanol‐containing assay buffer to adjust the final volume to 50 µL per well, after which 2 µL of lipase solution was added to each well and mixed thoroughly followed by incubation at 37 °C in the dark for 20 min, then 50 µL of triglyceride detection working solution was added to each well and mixed well for another incubation at 37 °C in the dark for 60 min before measuring the absorbance at 570 nm using a microplate reader (Synergy H4, Bio Tek, USA) to establish the standard curve and calculate the triglyceride concentration in the samples based on the standard curve.

### Cholesteryl Ester Level

The levels of cholesteryl ester in the hippocampus of mice were measured using the Amplex Red Cholesterol and Cholesteryl Ester Assay Kit (S0211S, Beyotime, China). Hippocampal tissues were homogenized with an isopropanol (G75885B, Tansoole, China) addition using a tissue homogenizer (Tissuelyser‐24L, Jingxin, China), followed by centrifugation at 12,000 g for 5 min at 4 °C, and the supernatant was collected. Samples were diluted at least five‐fold with detection buffer containing isopropanol. Standard curves for total cholesterol and free cholesterol were first established. Then, 30 µL of the diluted sample was added to the sample wells of a 96‐well plate, and the volume was adjusted to 50 µL with the corresponding detection buffer containing isopropanol. 50 µL of total cholesterol detection working solution or free cholesterol detection working solution was added to each well, mixed thoroughly, and incubated at 37 °C in the dark for 30 min. The absorbance at 570 nm was measured using a microplate reader (Synergy H4, Bio Tek, USA). The contents of total cholesterol and free cholesterol in the samples were calculated based on the standard curves, and the content of cholesteryl esters was obtained by subtracting the free cholesterol content from the total cholesterol content.

### Reverse Transcription and Real‐Time Quantitative PCR

Total RNA was extracted from mice brain hippocampus tissue by the trizol method (R401‐01, Vazyme Biotech, China) and reverse transcribed to produce complementary DNA by using HiScript II Q RT SuperMix for qPCR (+gDNA wiper) (R223, Vazyme Biotech, China). Step 1: template RNA 1 µg, 4 µL 4 × gDNA wiper Mix, RNase‐free ddH_2_O to 16 µL, incubated at 42 °C for 2 min. Step 2: added 4 µL 5 × No RT Control Mix to 20 µL, then incubated at 5 °C for 15 min and 85 °C for 5 s. Real‐time PCR was performed using 0.4 µL forward and 0.4 µL reverse primers, 1 µL cDNA, 10 µL ChamQ SYBR qPCR Master Mix (Q321, Vazyme Biotech, China), and 8.2 µl ddH_2_O. All the PCR primers employed are listed in **Table** **1**. The qPCR reaction was carried out on the LightCycler (LightCycler 480 II, Roche LifeScience, Switzerland).

**Table 1:** PCR primers employed in the present study.

Gene	Forward primer (5’ → 3’)	Reverse primer (5’ → 3’)
*Acadl*	GCATCAACATCGCAGAGAAA	GGCTATGGCACCGATACACT
*Acadm*	TTGAGTTGACGGAACAGCAG	CCCCAAAGAATTTGCTTCAA
*Acox2*	ATCTTGGCATGTTGGTGACA	CACTAGGCCGAAGACGAGAC
*Acsl3*	CAGCATTCAGCAAAACCTCA	CTGTTTCCACGGGAGTCAAT
*Angptl4*	GGAAAAGATGCACCCTTCAA	TGCTGGATCTTGCTGTTTTG
*Cpt1a*	CATGTCAAGCCAGACGAAGA	TGGTAGGAGAGCAGCACCTT
*Cpt2*	TCCTCGATCAAGATGGGAAC	GATCCTTCATCGGGAAGTCA
*Hmgcs2*	GGCTGTCAAAACAGTGCTCA	GCAATGTCACCACAGACCAC
*Plin4*	ACCACCAGAGACACCCAGTC	CCCATGGTTAGTGGCTCTGT
*Sorbs1*	TACCGAGCGATCGAAAGACT	GGCTGGACTTGAGTGAGGAG
*Atp6v0e1*	ATACCACGGCCTTACTGTGC	CAGAATTGCAATCAGCCAAA
*Dmxl1*	TAGCCAAAGCAGCCTTTCAT	TGCTTTACGCCACCTCTCTT
*Ctso*	CGCTGGCTAAATGAGACACA	CCAGCTCATCGCATCAACTA
*LAMP1*	GATGAATGCCAGCTCTAGCC	CTGGACCTGCACACTGAAGA
*ATG5*	GGAGAGAAGAGGAGCCAGGT	GCTGGGGGACAATGCTAATA
*ATG7*	TCCGTTGAAGTCCTCTGCTT	CCACTGAGGTTCACCATCCT
*p62*	AGAATGTGGGGGAGAGTGTG	TTTCCCGACTCCATCTGTTC
LC3	ATGTGGAAAAGCAGCTGTGG	GCCGGATGATCTTGACCAAC

### ChIP‐qPCR

The hippocampus was first crosslinked with 10 mL PBS containing 1% formaldehyde (G60216A, Tansoole, China) at 37 °C for 10 min, and the reaction was stopped with 1.1 mL Glycine Solution (10X) to a final concentration of 125 mm at room temperature for 5 min. Discarding the PBS and washing tissues with cold PBS containing 1 mm PMSF (BL1426A, biosharp, China), the tissues were then added 1 mL PBS (1 mM PMSF) to lyse tissues by using a homogenizer at a speed of 1000 rpm for 5 min, after that, RIPA lysis buffer (BL504A, biosharp, China) containing aprotinin (1 µL mL^−1^) and leupeptin (1µL mL^−1^) was used to resuspend cells and incubated on ice for 20 min. Next, genomic DNA was cut into fragments (200–1000 bp) by performing sonication (VCX 150, Sonics, USA) at 20% PAML with 12 rounds of 20 s on and 30s off. While the whole ultrasound process was performed on an ice‐water bath. Chromatin immunoprecipitation and DNA purification were finished by ChIP Assay Kit (P2087, beyotime, China) and PCR Clean Up Kit (D0033, beyotime, China). At the last moment, q‐PCR was performed to amplification of target gene sequence, and gel electrophoresis was used to the detection of ChIP products.

### Dual‐Luciferase Reporter Assay

N2a cells were incubated into a 24‐well plate for 24 h, and they were divided into four groups. 1) Double fluorescent reporter gene + Vec group, 2) Double fluorescent reporter gene + OE‐PPARA group, 3) Double fluorescent reporter gene + Vec + Cap group, 4) Double fluorescent reporter gene + OE‐PPARA + Cap group. First, 0.8 µg double fluorescent reporter gene plasmid or double fluorescent reporter gene plasmid + overexpression PPARA plasmid was dissolved into 50 µL Opti‐MEM (31985070, Thermo Fisher Scientific, USA) reduced serum medium, and 2 µL Lipofectamine 2000 (11668019, Thermo Fisher Scientific, USA) was also dissolved into 50 µL medium in the same way for 5 min. Then, these two solutions were mixed and incubated at room temperature for 20 min. Following the removal of the N2a cells culture medium and adding 400 µL Opti‐MEM reduced serum medium, the transfection mixture was added to the 24‐well plate for 100 µL of each. After 24 h, capsaicin was added to a concentration of 10 µm and was continued to culture for 24 h. Last, applying Dual Luciferase Reporter Gene Assay Kit II (RG029S, Beyotime, China) to lyse cells, the Renilla luciferase and Firefly luciferase relative light units were measured by Multi‐made reader (Synergy H4, Bio Tek, USA).

### V‐ATPase ELISA

According to the instructions of the V‐ATPase ELISA kit (FT‐P9S3328X, Fantaibio, China), the following operations were performed: the tissue of hippocampus was ground with PBS, and centrifuged at 3000 rpm for 10 min then the supernatant was taken, or at the time point of stable protein expression, the supernatant of the cells was collected and centrifuged at 3000 rpm for 10 min, so as to remove particles and polymers. The standard samples and samples were added to the microporous enzyme label plate, and then the sample diluent and the detection of antibody‐HRP were added in sequence. The reaction holes were sealed with the sealing plate membrane and incubated in the oven at 37 °C for 60 min. Substrate A and B were added after washing the plate, and incubated at 37 °C for 15 min in the dark. Finally, Optical density (OD) values of each hole were measured at 450 nm with a microplate reader (Synergy H4, BioTek, USA).

### Western Blotting Assays

All the primary antibodies (**Table** **2**) were used to detect PPARA, Tau pathology (pS262, pS214, pS396, pS404), Aβ pathology, autophagy‐lysosomal related proteins (LAMP1, ATG5, ATG7, LC3B, p62), glial cell markers (Iba1, GFAP), and HRP‐linked secondary antibody (anti‐rabbit or anti‐mouse IgG conjugated to horseradish peroxidase, 1:5000).

**Table 2:** Antibodies used in the Western blotting analysis and their properties.

Antibody	Specificity	Type	Dilution for WB	Source	Catalog number
β‐actin	β‐actin	Poly‐	1:5000	SAB	21338
PPARA	PPARA	Mono‐	1:1000	Proteintech	66826‐1‐Ig
PPARA	PPAR alpha	Poly‐	1:1000	GeneTex	GTX101098
CTSD	Cathepsin D	Mono‐	1:5000	abcam	ab75852
CTSB	Cathepsin B	Mono‐	1:500	zenbio	R381757
PLIN2	Perilipin‐2	Poly‐	1:5000	Proteintech	15294‐1‐AP
LAMP1	LAMP1	Poly‐	1:1000 (1:200 for IF)	abcam	ab24170
ATG7	ATG7	Poly‐	1:1000	SAB	38148
ATG5	ATG5	Mono‐	1:1000	SAB	44254
LC3B	LC3B	Poly‐	1:1000	abcam	ab48394
p62	SQSTM1	Mono‐	1:1000	Cell Signaling	23214S
4G8	β‐Amyloid_17‐24_	Mono‐	1:200 for IF	Biolegend	800708
Aβ	β‐Amyloid	Mono‐	1:1000	Cell Signaling	8243t
pS262	Phospho‐Tau (Ser262)	Mono‐	1:1000	SAB	11111
pS214	Phospho‐Tau (Thr214)	Poly–	1:1000	SAB	11109
pS396	Phospho‐Tau (Ser396)	Mono‐	1:1000	SAB	11102
pS404	Phospho‐Tau (Ser404)	Mono‐	1:1000	Bioss	Bs‐2392R
Iba1	Iba1	Poly‐	1:1000 (1:200 for IF)	abcam	ab178846
GFAP	GFAP	Mono‐	1:1000 (1:400 for IF)	Cell Signaling	3670S
Anti‐Mouse	Anti‐Mouse lgG		1:5000	Invitrogen	31430
Anti‐Rabbit	Anti‐Rabbit lgG		1:5000	Invitrogen	31460

Mono‐, monoclonal, poly‐, polyclonal.

### Immunofluorescence

Frozen sections with a thickness of 12 µm were used for immunofluorescence and subsequent quantitative statistics and calculations. Frozen brain sections were removed from the −80 °C freezer and reheated at room temperature for 30 min, and brain sections were washed by using PBS (G4202, Servicebio, China) three times for 10 min of each. PBS containing 0.5% TritonX‐100 (LA1604602, Sinopharm, China) was used to permeabilize brain sections for 30 min at room temperature, and washed with PBS three times for 5 min each, then applied 5% BSA (ST023, Beyotime, China) −0.5% TritonX‐100‐PBS to block 1 h at room temperature. Brain sections were incubated with anti‐LAMP1, anti‐4G8, anti‐Iba1, and GFAP primary antibody for 24 h at 4 °C (Table 2). The second day, the brain sections first reheated at room temperature for 1 h, and then washed with PBS for 10 min 3 times and incubated with donkey‐anti‐rabbit Alexa Fluor 594 (711‐545‐152, Jackson ImmunoResearch, USA) or donkey‐anti‐mouse Alexa Fluor 488 (715‐585‐150, Jackson ImmunoResearch, USA) or Alexa Fluor 594 donkey anti‐mouse (A21203, Invitrogen, USA) for 1 h at room temperature. For BODIPY staining, sections were incubated in PBS with BODIPY 493/503 1 µg mL^−1^ (D3922, Invitrogen, USA) for incubating 15 min at room temperature. Finally, sections were mounted by using antifade mounting medium for fluorescence (BL739A, Biosharp, China). Pictures were visualized by using Confocal laser scanning microscopy (LSM880, Carl Zeiss, Germany).

### Cell Culture and Treatment

Mouse neuroblastoma N2a cells (CL‐0168, Procell Life, China) were maintained in DMEM (C3113‐0500, VivaCell, Israel) containing 10% fetal bovine serum (BS1614‐109, BIOEXPLORER, USA) and 1% 100 × penicillin–Streptomycin solution (BL505A, Biosharp, China) at 37 °C in a humidified incubator with 5% CO_2_ (Thermo371, Thermofisher, USA). In the following in vitro experiments, N2a cells non‐treated as control groups were used, N2a cells treated with Aβ_1‐42_ (APA007, APeptides, China) at a concentration of 5 µm for 24 h as Aβ_1‐42_ groups, N2a cells treated with Aβ_1‐42_ (5 µm) for 24 h and then replaced the complete culture medium, adding capsaicin (T1062, TargetMol, China) at a concentration of 10 µm for treating another 24 h as Aβ_1‐42_ + Cap groups, application of lipofectamine 2000 (11668‐027, Thermofisher, USA) reagent to the transfection of N2a cells with Vector or siATP6V0E1 RNA or shPPARA plasmid according to the manufacturer's introduction, then treated with Aβ_1‐42_ and capsaicin are named Vector/siATP6V0E1/shPPARA + Aβ_1‐42_ + Cap groups. For the western blotting and real‐time quantitative analysis, N2a cells were planked in the 6‐well plates. Samples were all collected after 48 h of finishing transfection. The mouse‐specific siRNA of ATP6V0E1 was synthesized by OBiO Technology (Shanghai, China), the sense sequence is UCUGGUAUCUGAAGUAUCAUUTT, and the antisense sequence is AAUGAUACUUCAGAUACCAGATT. Mouse‐specific shRNA targeting PPARA was synthesized by Youbio Technology (Nanjing, China), and the targeting sequence is CCCTTATCTGAAGAATTCTTA.

### mCherry‐GFP‐LC3 Dual Fluorescence Indicator System

mCherry‐GFP‐LC3 (mRFP‐GFP‐LC3) plasmid, which was donated by Prof. Jianzhi Wang and Prof. Gongping Liu from Huazhong University of Science and Technology, is a fusion protein widely used for autophagy flux detection. mCherry protein is insensitive to the acidic environment of lysosome and is able to maintain red fluorescence dots, but GFP protein is unstable under the acidic conditions of the lysosomes, leading to the green fluorescent dots quenching. N2a cells were cultured in the 6‐wall plates with cell‐climbing tablets and maintained 12 h. mCherry‐GFP‐LC3 plasmid and Vector or siRNA of ATP6V0E1 or shRNA were transfected of PPARA plasmid at the same time, replacing the culture medium and treating with Aβ_1‐42_ (5µm) after 6 h of transfection, then adding capsaicin based on our previous protocol, cells were captured using a positive fluorescence microscope (Axio Imager Z2, Carl Zeiss, Germany).

### Immunofluorescence Staining of LysoTracker Red and BODIPY

LysoTracker Red (40739ES50, Yeasen Biotechnology, China), a red PH sensor, can selectively remain in slightly acidic lysosomes and can be used for lysosomal‐specific fluorescence staining of living cells. N2a cells were planked in confocal petri dishes (BS‐15‐GJM, Biosharp, China), and removed the culture medium then adding DMEM consisting of LysoTracker (0.5 µm) after 48 h of transfection, dark incubation for 45 min in an incubator, after that replaced the medium and added DMEM consisting of BODIPY 1 µg mL^−1^ (D3922, Invitrogen, USA) for 15 min at room temperature. Cells were captured by using Confocal laser scanning microscopy (LSM880, Carl Zeiss, Germany).

### Determination of Lysosomal PH

For confocal fluorescence imaging of LysoSensor Yellow/Blue DND‐160 (L7545, Thermo Fisher Scientific, USA) in N2a cells across different treatment groups, after completing transfection and drug treatment, the probe was diluted to 2 µm in DMEM. Cells were incubated in the pre‐warmed probe solution at 37 °C for 1 h, followed by PBS washing. Pre‐warmed DMEM was then added to the cells before image acquisition using a confocal microscope (LSM880, Carl Zeiss, Germany). Fluorescence images were captured at emission wavelengths of 450 ± 33 nm (blue fluorescence, representing less acidic lysosomal environments) and 510 ± 20 nm (yellow fluorescence, indicating more acidic lysosomal compartments).^[^ ^38^, ^60^ ^]^ Using Fiji software, the yellow and blue fluorescence dots were measured for each cell.

For absolute lysosomal PH measurement in N2a cells, cells were seeded in 96‐well plates and incubated with 2 µm LysoSenso Yellow/Blue DND‐160 (L7545, Thermo Fisher Scientific, USA) diluted in DMEM at 37 °C for 3–5 min, followed by PBS washing. Cells were then briefly rinsed with PH calibration buffers (PH 3–7) containing 10 µm monensin (S1753, Beyotime, China) and 30 µm nigericin (Y261764, Beyotime, China), and immediately incubated with 100 µL of respective PH standard buffers at 37 °C for 10 min. Fluorescence intensity was measured using a microplate reader (Synergy H4, Bio Tek, USA) with dual excitation/emission settings (Ex = 329 nm/Em = 440 nm and Ex = 384 nm/Em = 540 nm). A standard curve was generated by plotting the fluorescence intensity ratio (ex:329/380) against known PH values, enabling calculation of absolute lysosomal PH in test samples based on their measured fluorescence ratios interpolated against this calibration curve.

### Statistical Analysis

Data were analyzed using GraphPad Prism software 9 (GraphPad Software, Inc., La Jolla, CA, USA). Statistical analyses included the unpaired *t*‐test, one‐way analysis of variance (ANOVA) followed by Tukey's post hoc test, and two‐way ANOVA followed by Bonferroni's post hoc test.^[^ ^30^ ^]^ The data were expressed as mean ± SEM, and *p *< 0.05 were considered to be significant.

### Ethics Approval and Consent to Participate

The experimentation was authorized by the Animal Ethics Committee of Jiangnan University (JN. No20240315m0360701[100]). All experiments conducted with these mice adhere to the ethical considerations and animals' well‐being.

## Conflict of Interest

The authors declare no conflict of interest.

## Author Contributions

H.Y and J.C contributed equally to this work. H.Y., X.Z., D.J., and G.C. performed experimental design. H.Y., J.C., F.W., D.J., G.C., Y.Z., and J.D. contributed to the experimental methods. H.Y., J.C., F.W., Y.M., S.B., and X.Z. contributed to data analysis. H.Y., X.Z., D.J., and G.C. wrote the final manuscript.

## Supporting information



Supporting Information

## Data Availability

The data that support the findings of this study are available from the corresponding author upon reasonable request.
