# Supplementary material for: Capsaicin Alleviates Autophagy‐Lysosomal Dysfunction via PPARA‐Mediated V‐ATPase Subunit ATP6V0E1 Signaling in 3xTg‐AD Mice
Source: Adv Sci (Weinh). 2025 Jul 28;12(39):e02707. doi: 10.1002/advs.202502707 (PMC12533174; doi:10.1002/advs.202502707)
Supplement: Supplementary file 1 — Supporting Information [file ADVS-12-e02707-s001.docx]

**Supplementary data**

**Fig. Sl Body weight of 3xTg-AD mice vs 3xTg-AD+Cap mice receiving either normal diet or capsaicin diet over the treatment duration.** The 3xTg-AD mice were fed a diet supplemented with 0.01% capsaicin for four months. No significant changes were observed among the molding process among the two groups: 3xTg-AD *n*=8, and 3xTg-AD+Cap *n*=16. Two-way ANOVA test followed by Bonferroni’s post hoc test, ns, not significant.

3

2

1

4

**(A)**

**(B)**

**Fig. S2 The pattern diagram of Morris water maze and the residence time of the mice in different quadrants.** A) The water maze was equally divided into four parts and named after four quadrants, annular was a transparent circular platform with a diameter of 10 cm protruding 1.5 cm above the surface of the water and was placed in the center of the northwest (Session 2) quadrant. B) The time for 3xTg-AD mice to stay in the quadrant platform located was significantly less than that of WT mice during test, while capsaicin treatment increased the time in platform quadrant. WT, *n* = 16; 3xTg-AD, *n* = 8; 3xTg-AD + Cap, *n* =16. Two-way ANOVA test followed by Bonferroni’s post hoc test for B. Data were shown as mean ± SEM. *^*^P <* 0.05, ns, not significant.

**(B)**

**(A)**


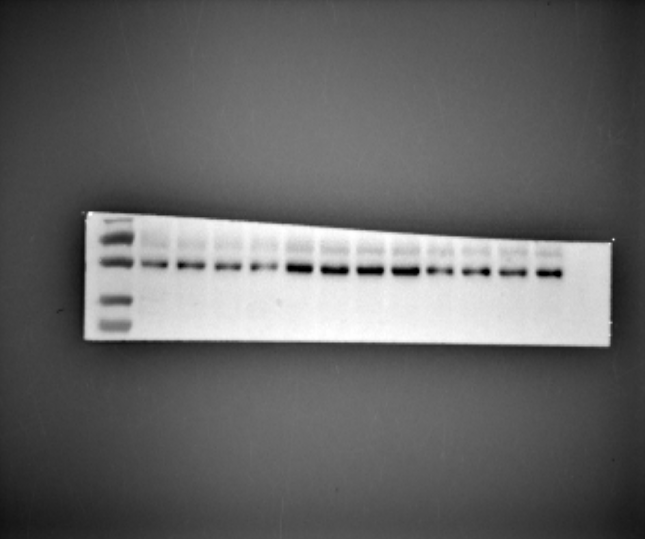


β-actin

-43


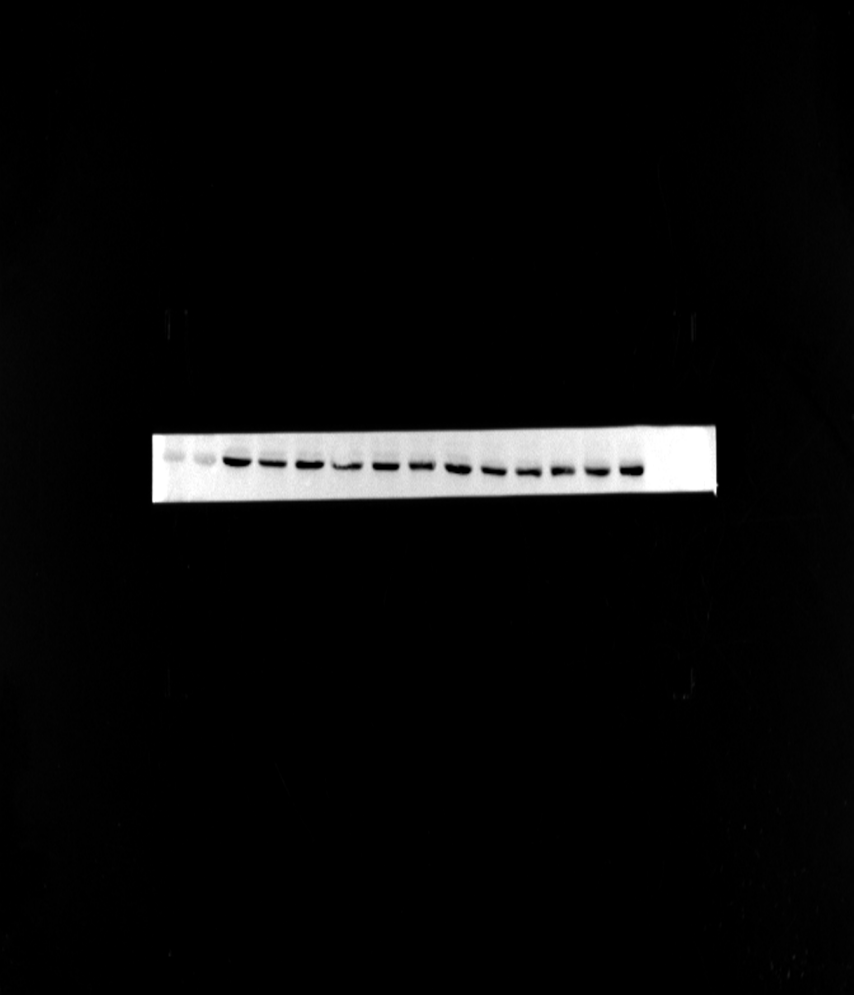


WT

3xTg-AD

3xTg-AD+Cap

**KDa**

PLIN22

-48

**Fig. S3** **Capsaicin significantly reduced the expression of lipid droplet-associated protein Plin2.** Western blotting analysis of Plin2 protein expression level in hippocampal tissues across three experimental groups A) and quantitative assessment B), *n*=4/group. One-way ANOVA followed by followed by Tukey’s post hoc test for B. Data were shown as mean ± SEM. *^*^P* < 0.05, *^**^P* < 0.01, *^***^P* < 0.001; *^****^P<* 0.0001; ns, not significant.

**(B)**

**(A)**

**Fig. S4 Capsaicin reduces lipid droplets accumulation in N2a-Αβ_1-42_ cells by enhancing lysosomal function.** A-B) LysoTracker Red and BODIPY immunofluorescence was used to evaluate lysosomal function and lipid droplets degradation. Quantification of the relative fluorescence density of LysoTracker, *n*=5/group A). Quantification of the number of BODIPY^+^ in 100x visual field, *n*=5/group B). One-way ANOVA followed by followed by Tukey’s post hoc test for A and B. Data were shown as mean ± SEM. *^*^P* < 0.05, *^**^P* < 0.01, *^***^P* < 0.001; *^****^P<* 0.0001; ns, not significant.

**(C)**

**(B)**

**(A)**


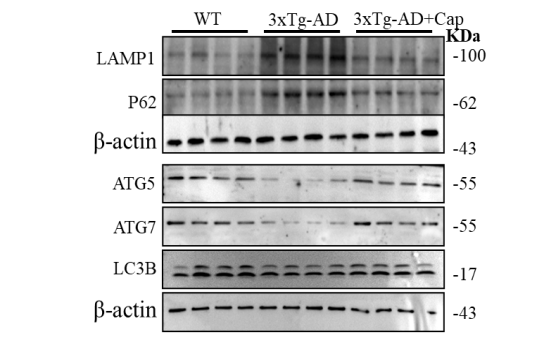


**Fig. S5** **Capsaicin promoted lipid degradation by enhancing the autophagy-lysosomal pathway.** A) The qRT-PCR analysis was used to analyze the gene levels of autophagy-lysosomal pathway in the hippocampus of WT, 3xTg-AD, and 3xTg-AD+Cap mice, *n*=4/group. B-C) The expression of autophagy-lysosomal pathway target genes in the hippocampus of 3xTg-AD mice fed with or without capsaicin and WT mice with western blotting analysis, *n*=4/group. One-way ANOVA followed by followed by Tukey’s post hoc test for A and C. Data were shown as mean ± SEM. *^*^P* <0.05, *^**^P* < 0.01, *^***^P* < 0.001; *^****^P<* 0.0001; ns, not significant.

**Fig. S6 Dmxl1 mRNA levels were elevated in Alzheimer's disease patients**. *Dmxl1* was elevated in the AD brain in GSE5281 dataset. Ctrl, *n*=13, AD, *n*=10. Unpaired t-test. Data were shown as mean ± SEM. *^*^P* <0.05, *^**^P* < 0.01, *^***^P* < 0.001; *^****^P<* 0.0001, ns, not significant.

**(A)**


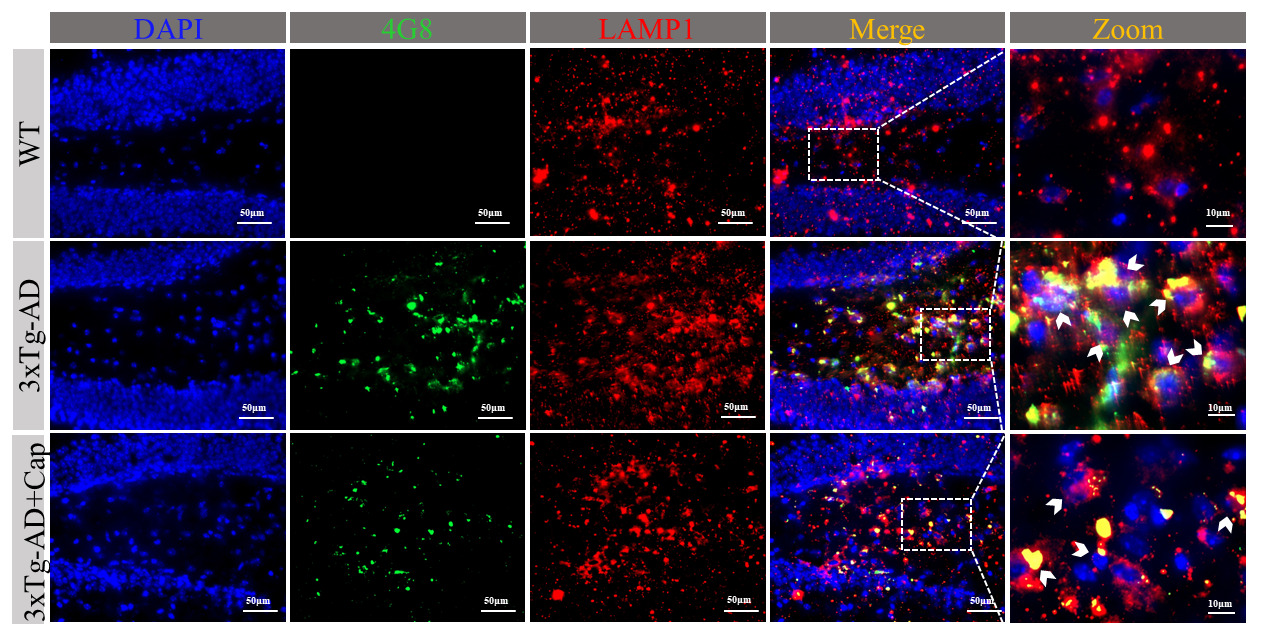


**(B)**

**(C)**

**(D)**

**(E)**

**(F)**

**(G)**


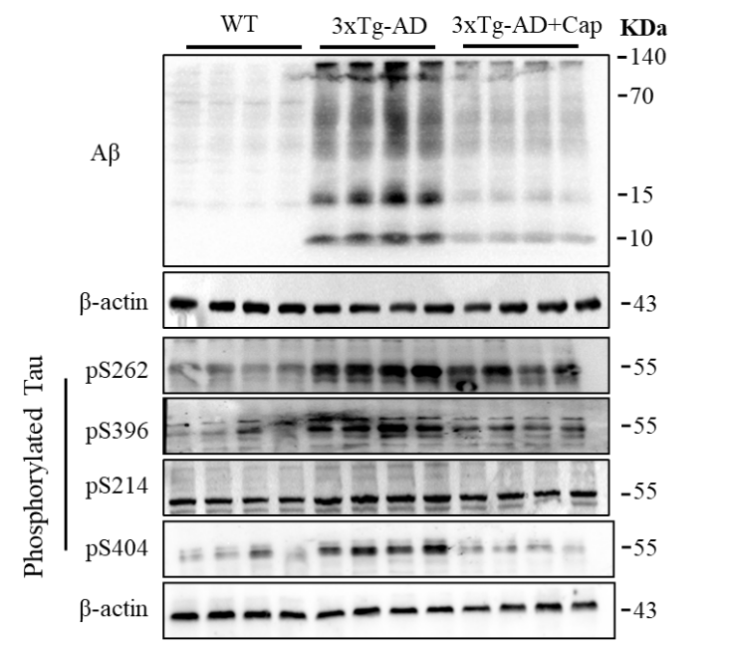

**Fig. S7 Capsaicin ameliorates AD-related pathology by enhancing the autophagy-lysosomal pathway.** A-E) Double-label immunofluorescence staining of Aβ-4G8 and LAMP1 in the DG area of hippocampus in 3xTg-AD mice treated with or without capsaicin and WT mice, representative confocal images of Aβ-4G8 and LAMP1 immunofluorescence co-labeling A), quantification of the percentage of area occupied by Aβ-4G8^+^ in visual field B), quantification of the fluorescence intensity of Aβ-4G8^+^ plaques area in visual field C), quantification of the fluorescence intensity of LAMP1^+^ D), quantification of the number of Aβ-4G8^+^ and LAMP1^+^ cells in visual field E), *n*= 3/group, and 5 visual fields/group were imaged. F-G) Examination of the changes of Aβ pathology and Tau pathology in the hippocampus of 3xTg-AD mice after treating with or without capsaicin and WT mice with western blotting analysis, *n*=4/group. One-way ANOVA followed by followed by Tukey’s post hoc test for B, C, D, E, G. Data were shown as mean ± SEM. *^*^P* <0.05, *^**^P* < 0.01, *^***^P* < 0.001; *^****^P<* 0.0001.

**(B)**

**(A)**

**
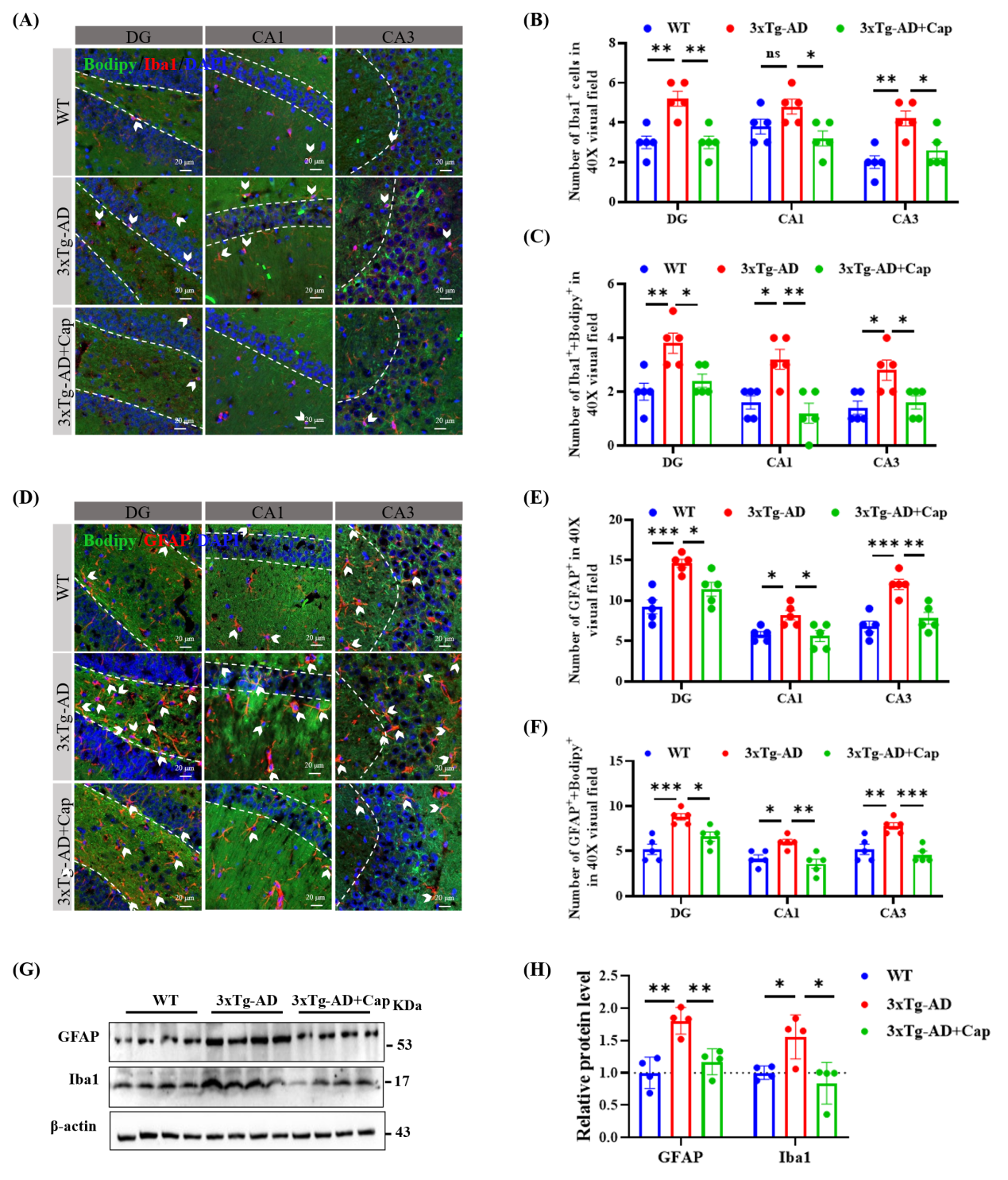
 Fig. S8 Capsaicin reduces inflammatory activation and lipid accumulation of microglia and astrocytes in 3xTg-AD mice****.** A-C) Characterization of the staining of BODIPY and Iba1 in the hippocampus of 3xTg-AD mice treated with or without capsaicin and WT mice via immunofluorescence; representative confocal images of BODIPY and Iba1 immunofluorescence co-labeling A), quantification of the number of Iba1^+^ cells in visual field B), quantification of the number of BODIPY^+^ and Iba1^+^ cells in visual field, *n*= 3/group, and 5 visual fields/group were imaged. C). D-F) Characterization of the expression of BODIPY and GFAP in the hippocampus of 3xTg-AD mice treated with or without capsaicin and WT mice via immunofluorescence representative confocal images of BODIPY and GFAP immunofluorescence co-labeling D). Quantification of the number of GFAP^+^ cells in visual field E). Quantification of the number of BODIPY^+^ and GFAP^+^ cells in visual field, *n*= 3 mice/group, and 5 visual fields/group were imaged. F). G-H) The relative protein levels change of GFAP and Iba1, *n*=4/group. One-way ANOVA followed by followed by Tukey’s post hoc test for B, C, E, F and H. Data were shown as mean ± SEM. *^*^P* <0.05, *^**^P* < 0.01, *^***^P* < 0.001, *^****^P<* 0.0001, ns, not significant.
